# A community‐based lung cancer rapid tissue donation protocol provides high‐quality drug‐resistant specimens for proteogenomic analyses

**DOI:** 10.1002/cam4.2670

**Published:** 2019-11-20

**Authors:** Theresa A. Boyle, Gwendolyn P. Quinn, Matthew B. Schabath, Teresita Muñoz‐Antonia, James J. Saller, Luisa F. Duarte, Laura S. Hair, Jamie K. Teer, Derek Y. Chiang, Rebecca Leary, Connie C. Wong, Alexander Savchenko, Angad P. Singh, LaSalette Charette, Kate Mendell, Gullu Gorgun, Scott J. Antonia, Alberto A. Chiappori, Benjamin C. Creelan, Jhanelle E. Gray, Eric B. Haura

**Affiliations:** ^1^ Department of Oncologic Science Morsani College of Medicine University of South Florida Tampa FL USA; ^2^ Anatomic Pathology Department H. Lee Moffitt Cancer Center & Research Institute Tampa FL USA; ^3^ Department of Thoracic Oncology H. Lee Moffitt Cancer Center & Research Institute Tampa FL USA; ^4^ Department of Ob‐Gyn New York University School of Medicine New York NY USA; ^5^ Department of Cancer Epidemiology H. Lee Moffitt Cancer Center & Research Institute Tampa FL USA; ^6^ Tumor Biology Department H. Lee Moffitt Cancer Center & Research Institute Tampa FL USA; ^7^ District 12 Medical Examiner's Office Sarasota FL USA; ^8^ Department of Biostatistics and Bioinformatics H. Lee Moffitt Cancer Center & Research Institute Tampa FL USA; ^9^ Novartis Institutes for BioMedical Research Cambridge MA USA; ^10^ Novartis Pharmaceuticals Corporation East Hanover NJ USA

**Keywords:** donation, heterogeneity, lung cancer, PD‐L1, rapid autopsy, resistance mutation, specimen quality

## Abstract

**Background:**

For the advancement of cancer research, the collection of tissue specimens from drug‐resistant tumors after targeted therapy is crucial. Although patients with lung cancer are often provided targeted therapy, post‐therapy specimens are not routinely collected due to the risks of collection, limiting the study of targeted therapy resistance mechanisms. Posthumous rapid tissue donation (RTD) is an expedient collection process that provides an opportunity to understand treatment‐resistant lung cancers.

**Methods:**

Consent to participate in the thoracic RTD protocol was obtained during patient care. When death occurred, tumor and paired non‐tumor, cytology, and blood specimens were collected within 48 hours and preserved as formalin‐fixed and frozen specimens. Tissue sections were evaluated with hematoxylin and eosin staining and immunohistochemistry (IHC) against multiple biomarkers, including various programmed death ligand 1 (PD‐L1) clones. Next‐generation sequencing was performed on 13 specimens from 5 patients.

**Results:**

Postmortem specimens (N = 180) were well preserved from 9 patients with lung cancer. PD‐L1 IHC revealed heterogeneity within and between tumors. An *AGK‐BRAF* fusion was newly identified in tumor from a donor with a known echinoderm microtubule‐associated protein‐like 4 to anaplastic lymphoma kinase (*EML4‐ALK*) fusion and history of anaplastic lymphoma kinase (ALK) inhibitor therapy. RNA expression analysis revealed a clonal genetic origin of metastatic cancer cells.

**Conclusions:**

Post‐therapy specimens demonstrated PD‐L1 heterogeneity and an acyl glycerol kinase to B‐rapidly accelerated fibrosarcoma (*AGK‐BRAF*) fusion in a patient with an *EML4‐ALK*–positive lung adenocarcinoma as a potential resistance mechanism to ALK inhibitor therapy. Rapid tissue donation collection of postmortem tissue from lung cancer patients is a novel approach to cancer research that enables studies of molecular evolution and drug resistance.

## Graphical abstract

We collected high‐quality primary and metastatic lung cancer specimens from donors after death by rapid collection in the community. Protein analysis of this tissue revealed discordant programmed death ligand 1 (PD‐L1) results at different cancer sites from the same patient; genetic analysis identified anaplastic lymphoma kinase (*ALK*) and B‐rapidly accelerated fibrosarcoma (*BRAF*) gene fusions in a patient with a history of ALK inhibitor therapy and a clonal genetic origin of metastatic cancer cells.

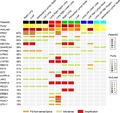

## 1 INTRODUCTION

Cancer research is underway to provide insights about the underlying mechanisms of cancer evolution and to develop biomarkers to predict responsiveness and resistance to mutation‐targeted and immuno‐oncology (IO) therapies. Such research is often hindered by a limited quantity of tissue collected at diagnosis and follow‐up time‐points. Rapid tissue donation (RTD) is the rapid collection of postmortem tissue with the specific goal of optimizing the high‐quality preservation of the specimens. The availability of such tissue after cancer progression provides information that may enable valuable insights into the causes of metastasis, genomic evolution, and mechanisms of targeted and IO therapy resistance.^1^, ^2^, ^3^, ^4^

The thoracic RTD protocol described here was developed with the hypotheses that rapid outpatient collection of tumor, metastases and paired non‐tumor specimens from recently deceased donors with lung cancer is feasible, and that these specimens could support meaningful research. The lung cancer population is often treated with immunotherapy or targeted therapy such that it is an ideal population for the study of immunotherapy and targeted therapy resistance. Although several other postmortem tissue collection studies have been described, few were dedicated to the collection of lung cancer tissues. Most RTD studies limit their tissue collections to the hospital environment which can extremely limit collection of lung cancer specimens since most individuals with lung cancer do not die in hospitals.^5^, ^6^, ^7^ Our community‐based collection approach with a protocol designed to request consent from patients in the clinic when they are doing well and collection over a wide geographical region (Hillsborough, Pinellas and Pasco counties, 2742 mi^2^) has preserved a large quantity of lung cancer specimens to enable research.^8^ Successful collection of tissue with the RTD protocol required a labor‐intensive effort to develop and implement a standardized protocol that is sufficiently agile to accomplish rapid tissue collection. Challenges we faced and overcame were the complexity of the ethics and logistics for rapid collection in the community and design of metrics to assess the quality of the donated tissue. The quantity and quality of tissue collected by this protocol demonstrates the feasibility of such community‐based collection of postmortem lung cancer tissue.

The hypothesis for this study was that we could use the outpatient collected RTD tissue to study lung cancer metastasis and evolution with proteogenomic studies. Herein, we describe successful collection of lung cancer tissue in the community from a tri‐county region and the use of this tissue to better understand the post‐therapy proteogenomic landscape of lung cancer.

## 2 MATERIALS AND METHODS

### 2.1 Lung cancer patients

**Figure 1:**
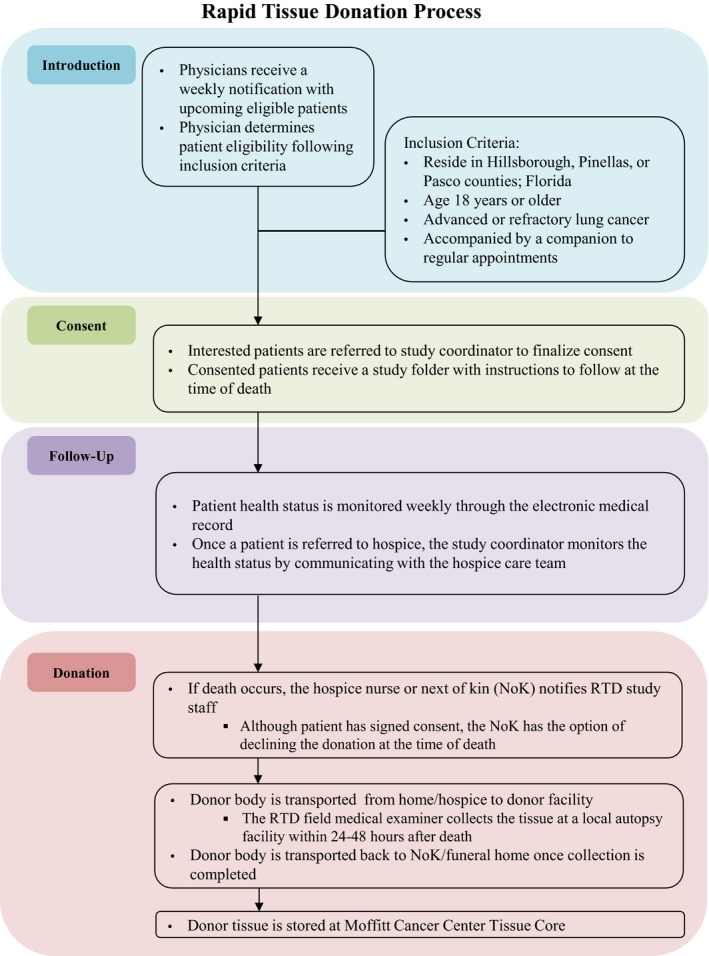
Flowchart overview of steps involved in the process of collection of tissue from donors participating in the rapid tissue donation project. In short, medical oncologists introduced the rapid tissue donation project to patients at an appropriate time. If interest was expressed from a patient, consent was subsequently requested. Posthumous tissue was rapidly collected by a medical examiner in the community at a facility as close as possible to the funeral service

The RTD logistics and eligibility criteria are depicted in Figure 1. The study and informed consent form was approved by the Institutional Review Board (IRB protocol 00014653; Advarra).

### 2.2 Specimen collection

Shortly after death (<48 hours), the study medical examiner (LSH) received consent documentation, storage containers, and a body map that represented primary and metastatic sites from review of clinical and radiologic information. Tissue specimens from primary tumor and metastatic sites, with matched non‐tumor tissue and blood, were collected from nine donors in a minimally invasive way and placed immediately in 10% neutral‐buffered formalin or liquid nitrogen. Specimens (N = 180) were preserved as either formalin‐fixed paraffin embedded (FFPE) blocks or frozen specimens.

### 2.3 Immunohistochemistry Analysis

Formalin‐fixed paraffin embedded and frozen tissue sections were stained with hematoxylin and eosin (H&E) and evaluated by a pathologist for tumor and histopathological quality. Formalin‐fixed paraffin embedded tissue with tumor was stained with programmed death ligand 1 (PD‐L1) immunohistochemistry (IHC) using the anti‐PD‐L1 rabbit monoclonal antibody clones, E1L3N^9^ and 28‐8 (Document S1, antibody information). All PD‐L1 stained slides were scored for percentage of tumor cell membrane staining with the tumor proportion score (TPS, scale 0%‐100%) with a positive TPS score cutoff ≥1% (Table S1). Exploratory IHC analysis with antibodies to Ki67, CD8, CD31, and pSTAT3 was performed with scoring as negative, low, medium, or high based on predefined criteria (Table S1). CD31 staining was used to calculate the intratumoral microvessel density for each tissue sample with the vascular hotspot method; pSTAT3‐stained slides were evaluated qualitatively.

### 2.4 Next‐generation sequencing analysis

Specimens were selected from patients 1, 3, 4, 6, and 7 for sequencing analysis. Specimens from patients 2 and 5 were not included due to the absence of tumor in specimens from these patients. Specimens from patients 8 and 9 were not included because they were collected after the sequencing studies were performed. Nucleic acid (DNA and RNA) was extracted from 13 tumor and 2 non‐tumor FFPE samples and analyzed for nucleic acid quantity (Qubit^®^) and quality (Agilent Genomic DNA and RNA ScreenTape assays^®^; Agilent 2200 TapeStation System).^10^, ^11^ DNA libraries from the 13 tumor specimens were generated using the TruSeq Nano Library Preparation kit (Illumina). Hybridization capture was performed with a customized Agilent SureSelectXT panel to enrich coding regions from 567 cancer‐related genes and select introns from 57 genes frequently rearranged in solid tumor cancers (Tables S2 and S3).^12^ Sequence reads were aligned with Burrows‐Wheeler Aligner Maximal Exact Matches (BWA‐MEM) software (https://arxiv.org/abs/1303.3997) to the hg19 human reference genome, marked for PCR duplicates with Picard (http://broadinstitute.github.io/picard/), and base quality scores were recalibrated with the Genome Analysis ToolKit.^13^, ^14^ Single nucleotide variants (SNVs) were called with MuTect,^15^ insertions and deletions (indels) with Pindel,^16^ translocations with Socrates,^17^ and tumor purity and copy number alterations with PureCN.^18^

RNA libraries from the 13 tumor specimens were prepared for RNAseq using the RNAseH protocol.^19^, ^20^ Briefly, the total RNA was depleted of ribosomal RNA, fragmented, converted to cDNA, and then a next‐generation sequencing (NGS) library was constructed using the TruSeq RNA v2 Library Preparation kit (Illumina). Sequence reads were aligned with STAR^21^ to the hg19 human reference genome, and read counts of coding regions were summarized by high throughput sequence (HTSeq) analysis^22^ using a human reference (Refseq) transcriptome. Principal components analysis (PCA) was performed with the read counts to assess the similarity of RNA profiles between tumor sites.

Additional sequencing of one FFPE sample for fusion confirmation was performed with the Illumina TST170 NGS platform and sequencing of nucleic acid from 11 frozen tumor tissue specimens was performed with the Agilent ClearSeq comprehensive cancer panel (Document S2).

## 3 RESULTS

### 3.1 Study population

Between November 2015 and November 2017, 21 lung cancer patients consented to the RTD study (Table 1) and 180 specimens, including tumor, paired non‐tumor, pleural effusion, pericardial fluid, and blood specimens, were retrieved from 9 donors, with the remaining 12 lung cancer patients still alive (Table S4). Tissue was successfully collected within 20 hours of death from 8 donors and at 41 hours from one due to additional unavoidable logistical complexities.

**Table 1:** Participant demographics

Number of consented Patients	Average age at enrollment	Average smoking history (pack years)	Histology			Sex	
			Adenocarcinoma	Small cell	Squamous cell	Male	Female
21	66	38	67%	19%	14%	43%	57%

### 3.2 Evaluation of H&E slides

**Figure 2:**
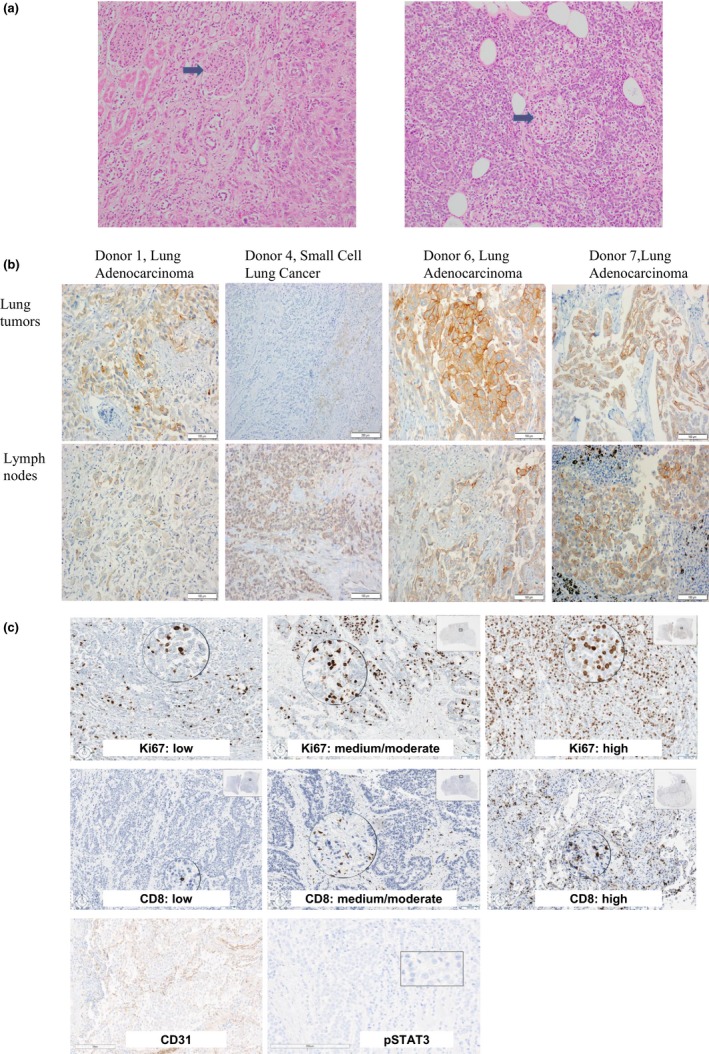
A, Hematoxylin and eosin stained slides at 200× magnification depicting well‐preserved kidney (on left, arrow indicates preserved glomerulus) and pancreas (on right, arrow indicates preserved pancreatic islet) from rapid tissue donation patient 1. B, Images of slides stained by immunohistochemistry with the E1L3N^®^ anti‐PD‐L1 rabbit monoclonal antibody from one representative lung tumor and one lymph node with metastatic lung cancer from donors 1, 4, 6, and 7 which had tumor with any positivity for PD‐L1 expression. C, Representative images of slides stained by immunohistochemistry with for Ki67, CD8, CD31, and pSTAT3

Histological evaluation of H&E stained slides revealed high‐quality tissue preservation with generally intact tissue architecture, distinct cancer cell nuclear staining, intact kidney glomeruli, tubules, and pancreatic islets (Figure 2A, kidney on left with arrow pointing to glomerulus, pancreas on right with arrow pointing to pancreatic islet).^23^, ^24^ Lung cancer was confirmed in 37 specimens collected from 7 patients (Table S5).

In two patients, patients 2 and 5, tumor was not identified in the postmortem tissue. Computed tomography review showed that patient 2 had only a thin rim of tumor surrounding necrosis that was consistent with a history of immunotherapy. Patient 5 had a small, 0.1 cm, lung lesion in a background of reactive changes and consolidated necrosis and metastatic lesions in the brain, but our current protocol limits brain tissue collection for cosmetic reasons. Thus, tumor collection from these two patients was complicated by small tumor amounts, obscuring changes in the surrounding tissue, and our protocol limitations on collecting brain tissue.

### 3.3 IHC analyses of protein expression

Immunohistochemistry evaluation was performed to assess the ability of RTD samples to support proteomic research. PD‐L1 IHC was initially performed on microscopically confirmed FFPE tumor tissue with the E1L3N antibody. Microscopic evaluation revealed distinct PD‐L1 membrane staining with a spectrum of PD‐L1 TPS scores from 0% to 95% (Table 3; Figure 2B). The differences in PD‐L1 TPS scores between primary and metastatic tumor sites within the same patients ranged from 0% (patient 3) to 55% (patient 6). PD‐L1 analysis on a subset of these tumor samples using a different PD‐L1 antibody, clone 28‐8, revealed similar TPS scores (Table 2). In Patient 7, PD‐L1 was positive during patient care (TPS score = 90%, 22‐C3 PD‐L1 antibody clone) and had PD‐L1 TPS scores in the postmortem tissue of ≥40% for two RTD tumor sites with the E1L3N and 28‐8 clones. Despite the overall similar PD‐L1 results in the RTD patients, if a 50% positivity cut‐off were used to guide immunotherapy, the results of PD‐L1 testing at different tumor sites would be discordant in 60% (3 of 5) and 20% (1 of 5) of patients tested with the E1L3N and 28‐8 clones, respectively.

**Table 2:** Collected case summaries

Case	Age	Smoking history	Histology	Treatment	Mutations	Total number of samples collected	Hours to collection	DIN	RIN	IHC and NGS performed
1	62	46 y	Adenocarcinoma	Pemetrexed/carboplatin	*KRAS G12V* (detected in post mortem tissue); Negative for *EGFR*	25	18	6.02	4.84	Yes
2	68	46 y	Small cell	Cisplatin/etoposide Carboplatin/etoposide atezolizumab Carboplatin/paclitaxel Carboplatin/etoposide	Negative for *FGFR*	15	41	—	—	No
3	63	46 y	Adenocarcinoma	Carboplatin/pemetrexed	‐	23	9	—	—	Yes
4	62	42 y	Small cell	Carboplatin/etoposide	‐	21	4	6.95	4.85	Yes
5	51	34 y	Small cell	None	‐	17	18	—	—	No
6	71	50	Adenocarcinoma	Nivolumab/Ipilimumab Erlotinib/MEK‐162	*KRAS G12D*; Negative for *EGFR *and *ALK*	20	5	7.65	5.85	Yes
7	70	None	Adenocarcinoma	Carboplatin/pemetrexed/bevacizumab nivolumab Crizotinib Alectinib	*EML4-ALK*; Negative for *EGFR*	15	13	6.73	4.05	Yes
8	73	50 y	Adenocarcinoma	Durvalumab/tremelimumab Carboplatin/pemetrexed	*KRAS* Q61H; Negative for *EGFR, BRAF, KRAS, PIK3CA, ALK, ROS1, RET*	25	19	—	—	No
9	51	None	Adenocarcinoma	Carboplatin/pemetrexed/bevacizumab nivolumab PBF‐509 Abemaciclib Carboplatin/Pemetrexed	*EGFR* exon 20 insertion; Negative for *KRAS* and *ALK*	19	15	—	—	No
						Total = 180	Average = 15.8	Average = 6.8	Average = 4.9	

Abbreviations: DIN, DNA integrity number; RIN, RNA integrity number; IHC, immunohistochemistry; NGS, next‐generation sequencing.

CD8, Ki67, CD31, and pSTAT3 IHC was performed in the same subset of RTD tumor samples (Tables 2 and 3; Table S1). Unequivocal antibody binding with specific staining patterns enabled classification into negative, low, moderate, and high CD8, Ki67, and CD31 expression groups; all tumor tissue was negative for pSTAT3 staining (Figure 2C). Levels of CD8, Ki67, and CD31 expression were similar between metastatic sites (Table 3), reflecting robust performance of IHC in the RTD specimens.

**Table 3:** Summary results of NGS and IHC biomarker analysis

Pt	Tissue	DNAseq				IHC					Clinical test results
		No of coding SNV	No of unique SNV	Oncogene	CHIP mutation	PD‐L1 (E1L3N) TPS (%)	PD‐L1 (28‐8) TPS (%)	CD8	Ki67	CD31	
1	Left lung	14	1	*KRAS* G12V 14%	*JAK2* E558N 47%	55	85	High	Low	43.3	Not evaluated
	Right lung	21	1	*KRAS* G12V 41%	*JAK2* E558N 55%	40	40	Med	Low	27.3	
	Mediastinal lymph node	21	1	*KRAS* G12V 19%	*JAK2* E558N 46%	70	90	Mod	Low	27.3	
	Liver	20	0	*KRAS* G12V 51%	*JAK2* E558N 48%	70	95	Med	Low	34.0	
3	Lung right lower lobe	12	1	*KRAS* G12V 55%	*TET2* P1723S 57%	0	0	High	Med	29.7	Not evaluated
	Lung right upper lobe	14	2	*KRAS* G12V 49%	*TET2* P1723S 41%	0	0	Med	Low	91.7	
4	Right lung	16	2	*KEAP1* E441* 87%, *TP53* N239D 80%		0	0	Low	high	34.7	Not evaluated
	Pericardial lymph node	13	0	*TP53* N239D 79%		10	0	Med	high	52.3	
	Liver	15	2	*KEAP1* E441* 45%,* TP53* N239D 41%		0	0	Low	High	24.7	
6	Lung left lower lobe	7	1	*BRCA2* R2034C 50%, *KRAS* G12D 3%		95	95	High	Med	34.7	*KRAS*‐G12D positive, *EML4-ALK* negative, *EGFR* negative
	Lymph node	20	14	*BRCA2* R2034C 44%, *KRAS* G12D 10%		40	70	High	Low	32.7	
7	Lung left hilar	10	1	*SMARCA4 *R1135Q 13%, *EML4-ALK* 2.8%		40	60	High	Low	27.3	*EML4-ALK* positive, *EGFR* negative, PD‐L1 positive
	Mediastinal lymph node	9	0	*SMARCA4* R1135Q 6%, *EML4-ALK* 2.7%		70	90	High	Med	14.7	

Abbreviations: IHC, immunohistochemistry; NGS, next‐generation sequencing; SNV, single‐nucleotide variant; CHIP, clonal hematopoiesis of indeterminate potential; PD‐L1, programmed death ligand 1; TPC, tumor proportion score.

Two of five patients with PD‐L1 IHC results had prior immune checkpoint inhibition (ICI) treatment (Table 2, Patients 6 and 7). Post‐ICI initiation, patient 6 had stable disease at 70 days, and progression with new left lung lesions at 168 days. Patient 7 had stable lesions in the left lung and adrenal gland at 70 days, but a 30% larger liver lesion and ICI was discontinued due to ICI‐induced pneumonitis. In both patients, postmortem tumor had PD‐L1 TPS scores ≥40% and a high density of intratumoral CD8 positive lymphocytes, consistent with cellular immune activation.

### 3.4 Nucleic acid extraction and sequencing from frozen tissue

The frozen tissue mean weight from 81 specimens from the first 8 patients was 430 mg (range 67‐850 mg). Nucleic acid was extracted from 15 tumor specimens from the first four patients with >200 mg weights. Average DNA and RNA yields were 14.8 μg (2.1‐26.6 μg) and 14.4 μg (3.4‐23.8 μg), respectively. Assessment of DNA and RNA integrity numbers (DINs and RINs, respectively), numbers that represent the quality or integrity of the nucleic acid, revealed average DINs and RINs of 6.5 (3.0‐8.0) and 4.9 (3.8‐6.9), respectively. A qualitatively small decrease in DNA and RNA quality was observed with longer interim times between death and tissue collection (Figure S1). NGS was performed on 11 DNA specimens from two patients with the Agilent ClearSeq Comprehensive Cancer panel and sequence quality metrics revealed high quality with approximately 25 million reads per sample, 99% of reads mapped and properly paired, median average coverage per targeted base of 730x (570x‐1220x), and duplicate rates less than 30%.

### 3.5 NGS‐based DNA and RNA analyses from FFPE tissue

DNA‐seq with a customized 567‐gene Agilent SureSelectXT panel was performed on 13 tumors from FFPE tissue sections from patients 1, 3, 4, 6, and 7. Tissues from patients 2 and 5 were not included because of the absence of tumor in the collected specimens, and patients 8 and 9 were not included in the molecular analysis because their tissue was collected later. The quality metrics for sequencing coverage demonstrated high overall coverage with ~87% of reads mapped to the targeted genes (Table S6). The median average coverage per targeted base was 547x (448x‐816x), and median duplicate rate was 35% (15%‐45%). The estimated tumor purity, as estimated by PureCN,^18^ varied from 16% to 73%.

**Figure 3:**
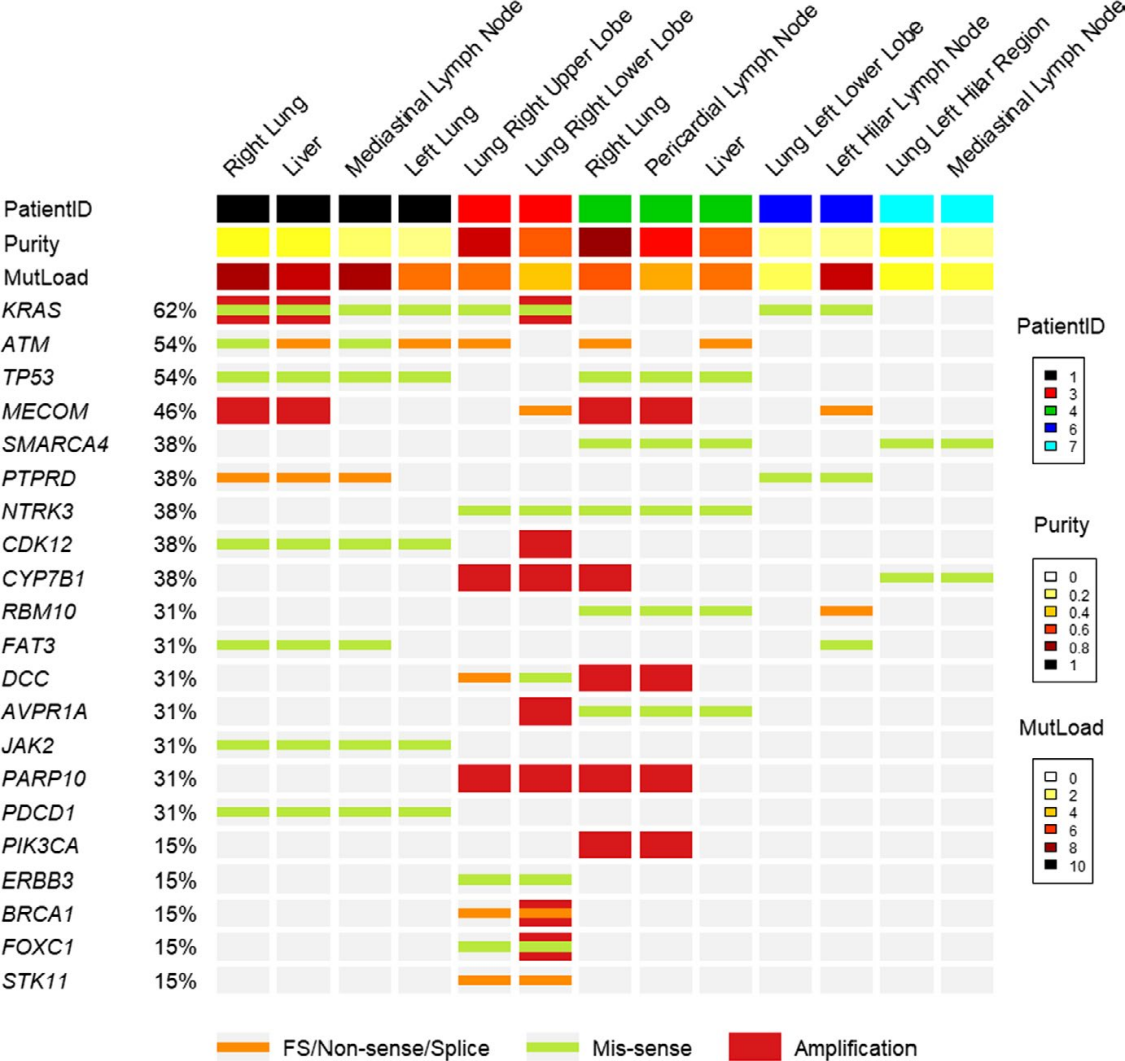
Genomic heterogeneity of mutations among multiple tumor sites. The columns represent specimen results from sequencing 13 specimens from 5 donors with a customized 567‐gene Agilent SureSelectXT panel. Each column represents one specimen with the top bar color representing which donor the specimen is from: 1 (black), 3 (red), 4 (green), 6 (dark blue), and 7 (light blue). For each gene on the left, there is an orange bar if a frame shift (FS) mutation, nonsense mutation, or splice variant is identified, a green bar if a missense mutation is identified, and a red bar if amplification is identified in that gene. If no colored bar is present (gray background), the specimen was negative for mutations in that gene. Abbreviations: FS, frameshift; purity, calculated tumor percentage; MutLoad, mutation load (number of mutations identified)

The vast majority of genomic alterations were shared among multiple tumor lesions from the same patient, suggesting that the tumor from the disseminated metastatic sites all originated from a single founder clone (Figure 3; Table S7; Figure S2A‐E). For example, patients 1, 3, and 6 all had KRAS missense mutations in all tumor sites tested. In patient 3, complex aneuploidy of chromosome 8q was among the changes identified in both tumor sites. In patient 4, broad level chromosomal changes were identified in all three sequenced lesions. In patient 6, *KRAS* G12D was identified during patient care, concordant with the postmortem genetic results. In this patient, several genetic changes identified at low allele frequency in the metastatic lymph node tissue were not detected in the primary lung cancer, possibly due to tumor evolution, but could also be due to low tumor cellularity (<30%) in the lung cancer specimen with a higher risk for false negative results.

Patient 7 had an echinoderm microtubule‐associated protein‐like 4 to anaplastic lymphoma kinase (*EML4‐ALK*) gene fusion initially identified by fluorescence in‐situ hybridization during clinical care. This patient received 1st line chemotherapy prior to the *ALK* fusion detection, 2nd line immune checkpoint inhibition (ICI), and then two lines of ALK inhibitor therapy (Table 2). Genetic testing of the postmortem lung tumor and metastatic lymph node tissues revealed not only the *EML4‐ALK* fusion but also a previously unknown in‐frame acyl glycerol kinase to B‐rapidly accelerated fibrosarcoma (*AGK‐BRAF*) gene fusion. In this fusion, the 3′ end of exon 2 of the *AGK* gene is fused with the 5′ end of exon 8 of the *BRAF* gene. This results in a chimeric protein in which the *AGK* promotor through exon 2 is intact and reads into exon 8 of *BRAF,* with the *BRAF* kinase domain remaining intact. Two nucleotides for codon 34 are at the end of *AGK* exon 2, and the third‐position nucleotide is in exon 3. The fusion causes a missense change of codon 34 from TGT (cysteine) to TGG (tryptophan). The *BRAF* sequence continues in frame, with the wild‐type sequence encoding the BRAF protein from exon 8 to the end. These fusions were both confirmed with testing of a post‐ALK inhibitor pericardial tumor biopsy with the Illumina TST170 NGS platform.

**Figure 4:**
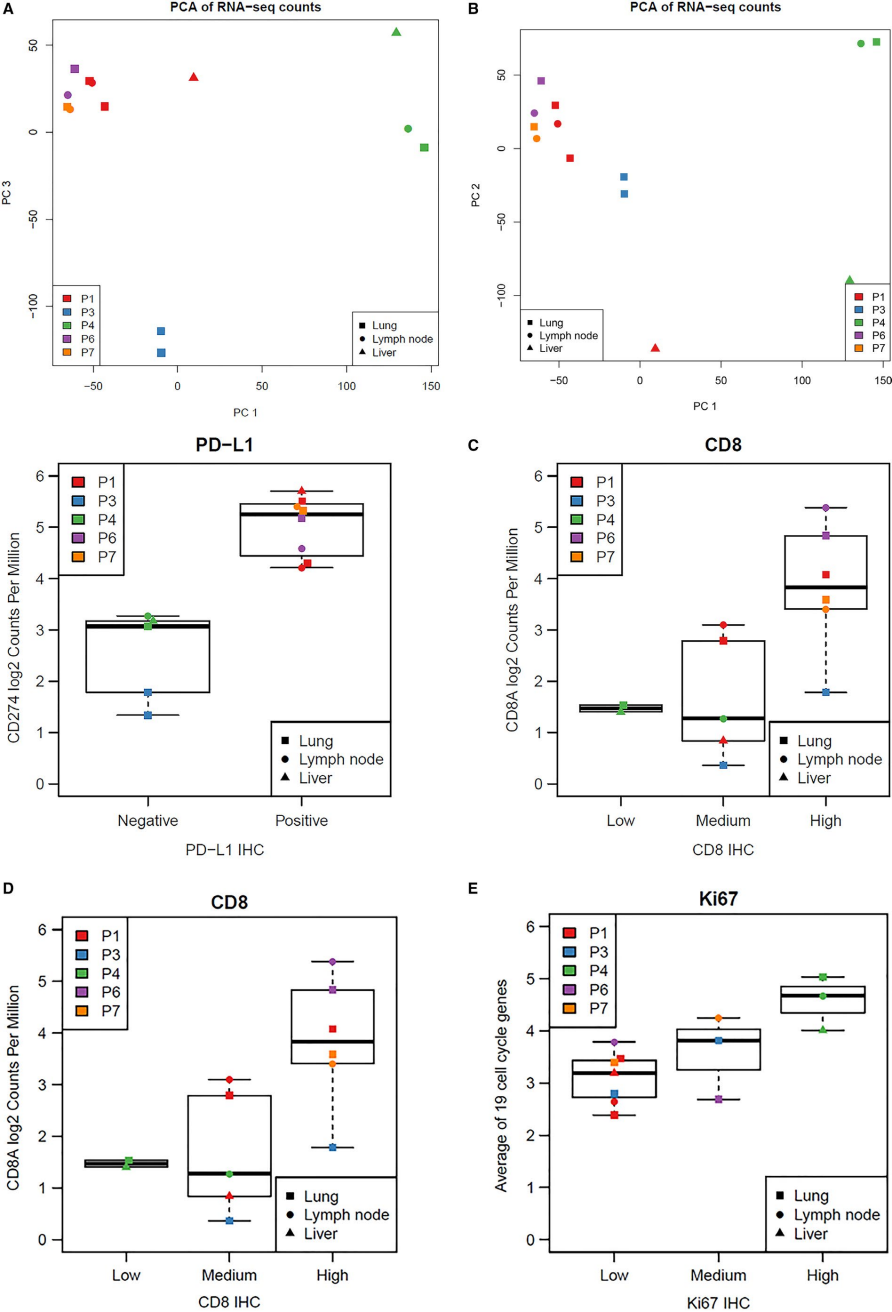
Compilation of RNA Results. A, Global similarity of RNA profiles among multiple tumor sites. The first and third principal components are shown for the normalized log2 RNA‐seq counts per million for all genes. A, The third principal component is shown instead of the second as this corrects for elevated levels of liver‐specific transcripts. B, The second principal component is shown instead of the third to show results without correction for elevated levels of liver‐specific transcripts. C‐E, Concordance between RNA‐seq and IHC for PD‐L1, CD8, and Ki‐67. The vertical axis denotes the normalized log2 RNA‐seq counts per million for the indicated genes. The horizontal axis denotes the final categorical result for PD‐L1, CD8, and Ki‐67 expression by immunohistochemistry analyses as outlined in Table S1. PD‐L1 immunochemistry data was generated using the PD‐L1 antibody clone 28‐8 with ≥1% tumor proportion score considered as “Positive”

RNA‐seq produced estimated library sizes greater than 10 million sequences and a median of 125.9 million (15.1‐287.0 million) for all 13 tumor sites tested (Table S6). As expected from a total RNA protocol after ribosomal depletion, the median percentage of coding bases was 15.4% (11.6%‐19.3%). The sequence duplication rates had a median of 12.2% (7.9%‐48.6%). PCA revealed potentially confounding elevated levels of several liver‐specific transcripts (Figure S3) in sequence from the liver biopsies. A liver‐specific signature of 21 genes (Document S3) and linear regression was used to identify liver‐specific genes with high shared expression to correct the PCA for the liver background (Figure 4A, with correction; B, without correction). The corrected PCA revealed that tumors from the same patient were more similar to each other than tumors from other patients. Of note, the only patient with a diagnosis of small‐cell lung cancer and RNA data (Patient 4) had RNA profiles from two tumor sites that were similar to each other and distinct from the other NSCLC tumors with higher levels of several mucins and surfactant proteins (MUC4, MUC5B, MUC6, SFTPB).

Next, PD‐L1, CD8, and Ki67 RNA expression levels per RNA‐seq were compared with IHC protein expression levels in matched RTD FFPE specimens (Figure 4C‐E). Specimens with high and low PD‐L1 (also known as CD274) gene and protein expression levels clustered together. The gene expression and IHC levels of CD8 were similar, albeit there was more overlap of RNA‐seq expression levels in the low and medium IHC categories. The average read counts from a cell cycle signature of 19 genes (Document S3) was generally concordant with Ki67 IHC scores. A possible explanation for the overlap in the low and medium expression groups for CD8 and Ki67 is that the IHC assessment was done in the intratumoral compartments only, whereas RNA‐seq analysis was performed using the whole tissue sample.

## 4 DISCUSSION

We used tissue collected through a novel lung cancer RTD program that enabled expedient collection of 180 postmortem specimens to describe the proteogenomic landscape of lung cancer and potential mechanisms of resistance to targeted therapy.

Logistical challenges to collection of post‐therapy lung cancer specimens included communication with hospice care facilities,^25^ locating autopsy facilities in the community, and identifying tumor in cases with tumor obscured by necrosis, consolidated atelectasis, and reactive changes. For two cases, in which no tumor was identified in the postmortem specimens, premortem tumor specimens were available. RTD programs benefit from having a plan to include premortem specimens collected during routine patient care, to be used in addition to the postmortem tissue, for research. Tissue was generally well‐preserved, consistent with reports by other rapid postmortem tissue donation studies.^5^, ^6^, ^7^ The specimen pool and value of the research specimens was optimized by the inclusion of premortem clinical care specimens associated with RTD cases, which allowed comparison of histologic and molecular findings between premortem and RTD‐collected postmortem specimens. The additional collection of premortem specimens into the RTD protocol allows analysis of premortem tissue when postmortem tissue yield or quality is compromised.

The successful performance of IHC staining with multiple antibodies, including PD‐L1, CD8, Ki67, and CD31, confirmed adequate preservation of protein antigenicity in the RTD tissue. The pSTAT3 staining was negative in all tested specimens, suggesting that phospho‐epitopes may be too labile for accurate testing in postmortem specimens. In general, the protein expression of PD‐L1, CD8, Ki67, and CD31 by IHC analysis was consistent between tumor sites within the same patients. However, a difference in PD‐L1 TPS scores of up to 55% between different tumor sites in the same patient was observed, consistent with previous reports of PD‐L1 heterogeneity in lung^26^ and other cancers.^27^ This means that with a cut‐off of ≥50% PD‐L1 TPS for positivity (the cut‐off for the companion diagnostic PD‐L1 assay for pembrolizumab), 20%‐60% of individuals in this study would have a different final PD‐L1 result if different tumor sites were tested. This illustrates the importance of interpreting PD‐L1 results with caution with the implication that 20%‐60% of patients might not be eligible for immunotherapy based on testing of one tumor site, but would be eligible based on testing of a different tumor site. PD‐L1 heterogeneity may also explain why some patient tumors that initially respond to anti‐PD‐L1 therapy progress later.

Several studies have concluded that frozen postmortem tissue can generate useful sequence and expression data, even with relatively lower DINs and RINs (<5).^6^, ^7^ Likewise, we demonstrated successful comprehensive genomic profiling from frozen and FFPE RTD lung cancer tissue. The primary and metastatic tumor sites in the same patients had similar genetic profiles, consistent with a study of postmortem lung cancer specimens that demonstrated a shared set of genomic activating mutations in primary and metastatic tumors from the same patients.^28^ In contrast, a study by the TRACERx consortium for Stage I‐III lung cancers reported more evidence of subclonal heterogeneity between different tumor areas with whole‐exome sequencing.^29^ This discordance might be explained by a difference in assays with the whole‐exome sequencing having greater coverage than the cancer‐focused targeted panel we used which covers approximately 3% of all protein‐coding regions.

An *AGK‐BRAF* fusion was newly identified in postmortem tumor specimens from a donor with a known *EML4‐ALK* fusion and resistance to ALK inhibitor therapy. Activated BRAF can promote oncogenesis by activating downstream MEK‐ERK signaling, and the *AGK‐BRAF* fusion has been identified previously in lung and other cancer types.^30^, ^31^, ^32^ This is a compelling finding in that if this *AGK‐BRAF* fusion had been detected during clinical care, the result may have informed clinical management with consideration of BRAF targeted therapy.

Principal components analysis of the RNA transcriptome revealed that multiple tumor sites from the same patient were more similar to each other than tumors from other patients. This finding agrees with a different study which concluded that the transcriptome of lung cancer lesions from the same patients cluster together with global unsupervised clustering analysis.^33^ The RNA‐seq data was concordant with PD‐L1, CD8 and Ki67 protein expression (Figure 4C‐E), underscoring the high quality of the transcriptomic and protein data.

In summary, an outpatient‐based RTD protocol with collection of high‐quality postmortem tissue across primary and multiple metastatic sites from advanced lung cancer patients in the community is feasible and enables research. The performance of IHC, DNA, and RNA sequencing on the collected FFPE and frozen tissue was reliable and supports the high value of postmortem tissue for cancer research. Differences in PD‐L1 expression at different tumor sites with 20%‐60% of patients “negative” at one tumor site and “positive” at another site urges caution in the interpretation of PD‐L1 results for immunotherapy. The identification of an *AGK‐BRAF* fusion as a potential resistance mechanism to ALK inhibitor therapy bears further investigation. Expanded evaluation of the immune microenvironment or whole‐exome sequencing to identify more passenger mutations may lead to additional insight regarding tumor metastasis, evolution, and mechanisms of resistance to immune checkpoint and/or targeted therapy.

## CONFLICT OF INTEREST

None of the authors associated with Moffitt Cancer Center claim a conflict of interest for this study. The authors associated with Novartis have a general conflict of interest due to the nature of their employment with a pharmaceutical company. Dr. Schabath is an Associate Editor for Cancer Medicine, and the authors confirm that this does not alter their adherence to Cancer Medicine Editorial policies and criteria.

## AUTHOR CONTRIBUTIONS

GPQ, MBS, TM‐A, SJA, AAC, BCC, JEG, EBH, LFD, and EBH conceptualized the RTD program, including the patient consent process. TAB, QPQ, MBS, TM‐A, JKT, DYC, RL, CCW, AS, APS, LC, KM, GG, and EBH performed data creation and formal analysis. All authors contributed to the writing and editing of the manuscript.

## Data Availability

The data that support the findings of this study are available from the corresponding author upon reasonable request.
