# Supplementary material for: A community‐based lung cancer rapid tissue donation protocol provides high‐quality drug‐resistant specimens for proteogenomic analyses
Source: Cancer Med. 2019 Nov 20;9(1):225–37. doi: 10.1002/cam4.2670 (PMC6943158; doi:10.1002/cam4.2670)

A Community Based Lung Cancer Rapid Tissue Donation Protocol Provides High Quality Drug Resistant Specimens for Proteogenomic Analyses

Supplemental Figures Legends

Supplemental Figure 1. The time in hours elapsed between death and tissue collection was plotted against average DNA or RNA integrity (DIN and RIN) score per rapid tissue donation case (N=4 cases).

Supplemental Figure 2, A-E. Chromosome plots for Patient 1 (2A), Patient 3 (2B), Patient 4 (2C), Patient 6 (2D), and Patient 7 (2E) depict genetic changes at the chromosomal level by chromosome. For example, in patient 3, complex aneuploidy of chromosome 8q (including *MYC*), as well as focal deletions in chromosomal regions 4p16 (*FGFR3*) and 9q34, was identified in both tumor sites. In patient 4, broad level chromosomal changes were identified in all 3 sequenced lesions, including arm-level gains of chromosomes 3q, 5p, 8q, and 18q and arm-level losses of chromosomes 5q, 10, and 17p. In patient 6, the lymph node metastasis harbored several focal high-level amplifications in chromosome 12q15 (including *MDM2*) and chromosome 14q13 (near *NKX2-1*) that were not detected in the lung tumor sites.

Supplemental Figure 3. Principle components analysis based on read counts from RNA-seq were used to assess the global similarity of RNA profiles by tumor site. Two liver metastatic lesions from patients 1 and 4 were more similar than the other lesions from the same patient, consistent with elevated levels of several liver-specific transcripts.

Supplemental Figure 1

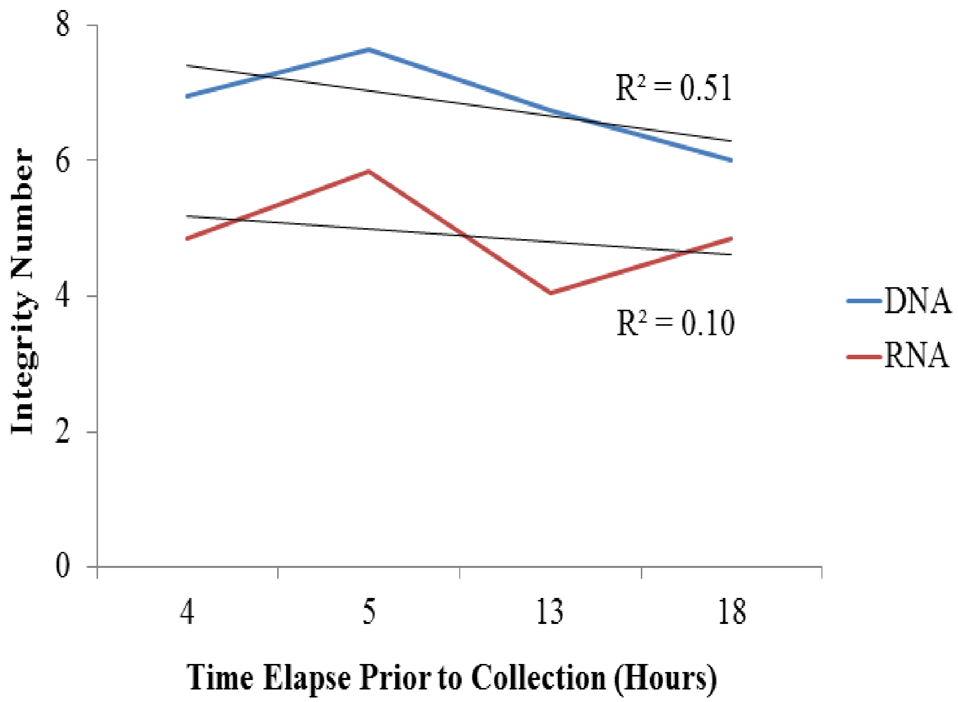

Supplemental Figure 2A (Patient 1: Left Lung)

LIB.021180pc3.1

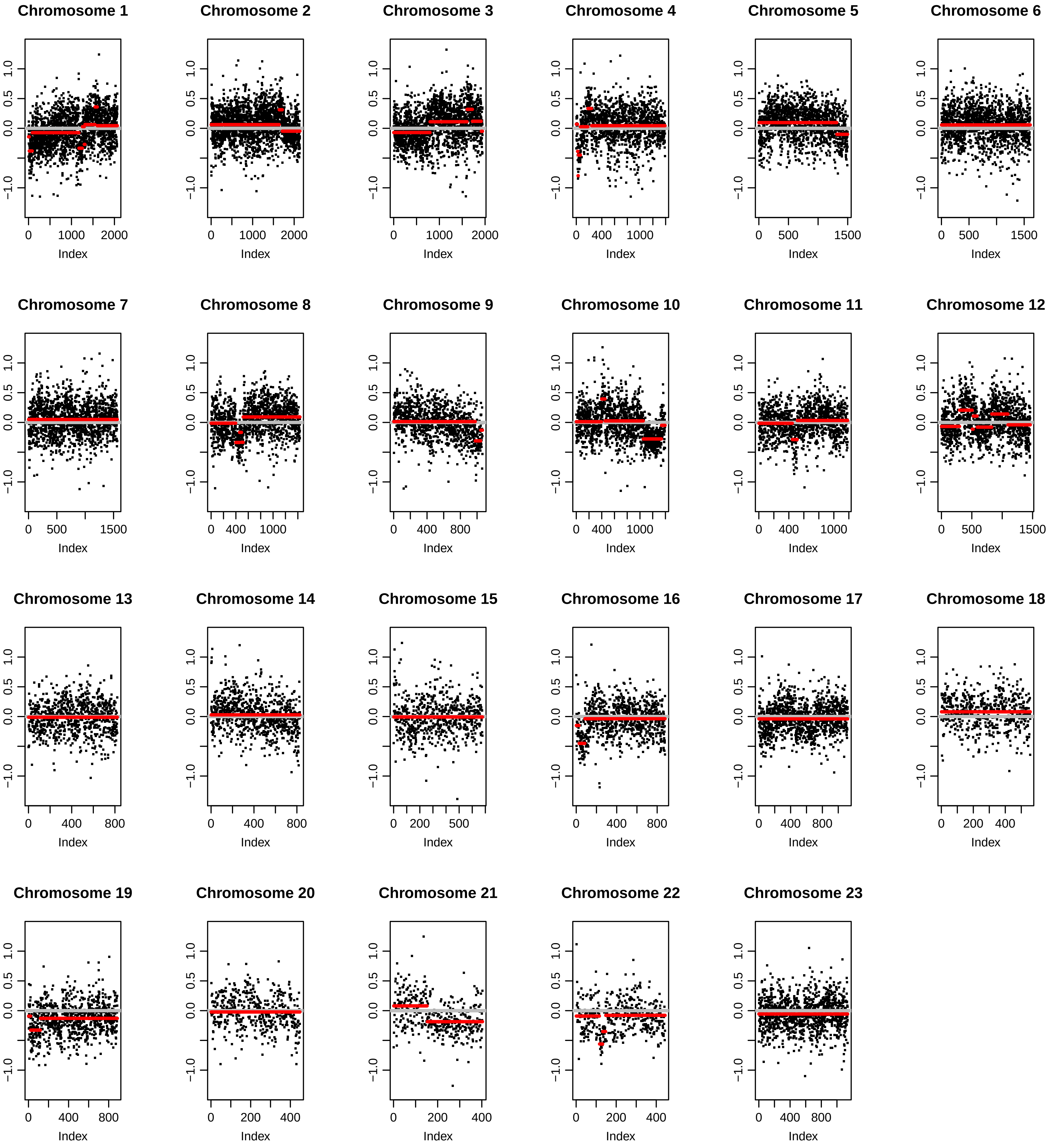

Supplemental Figure 2A (Patient 1: Right Lung) **LIB.021183pc3.1**

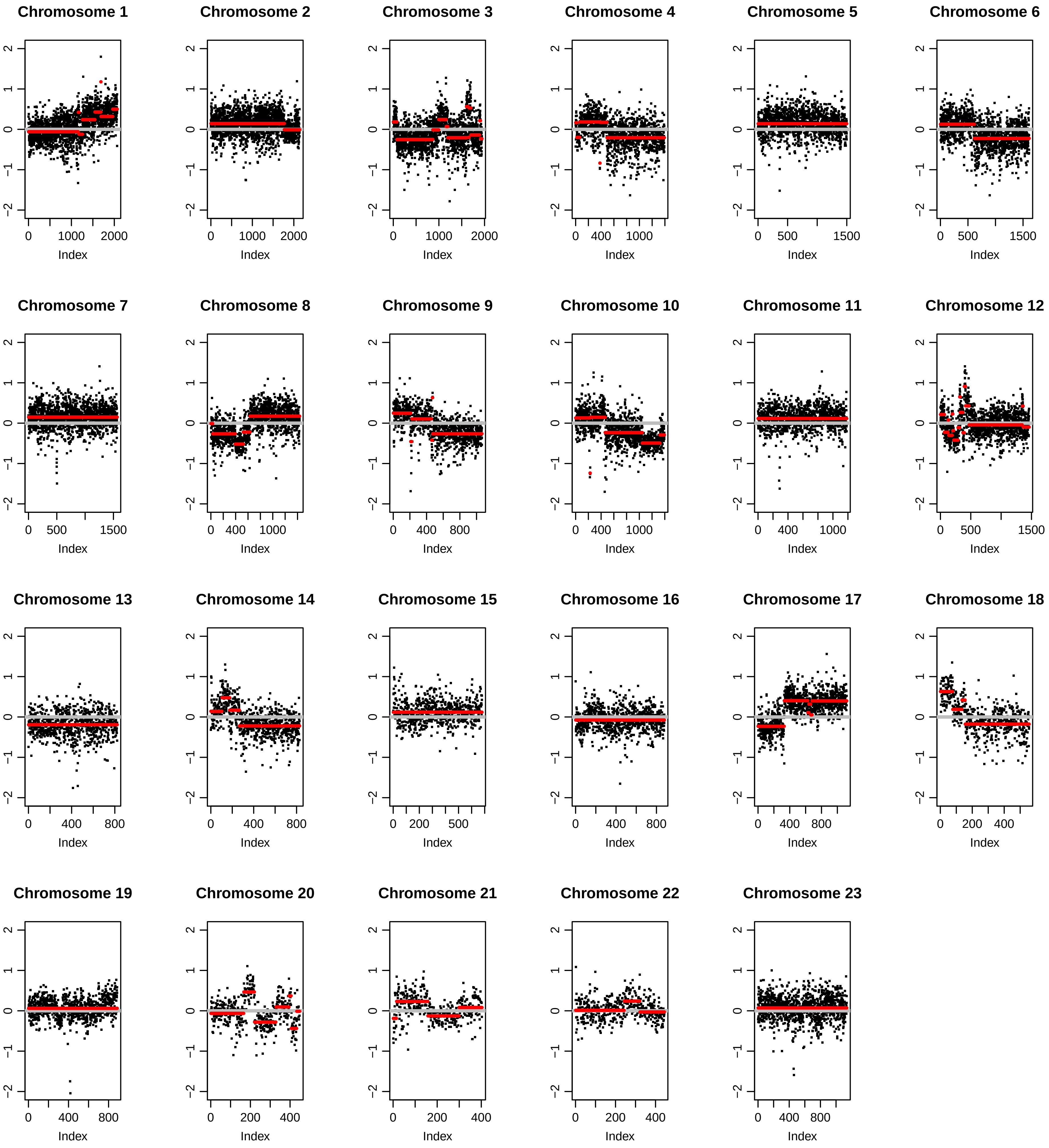

Supplemental Figure 2A (Patient 1: Lymph Node) **LIB.021179pc3.1**

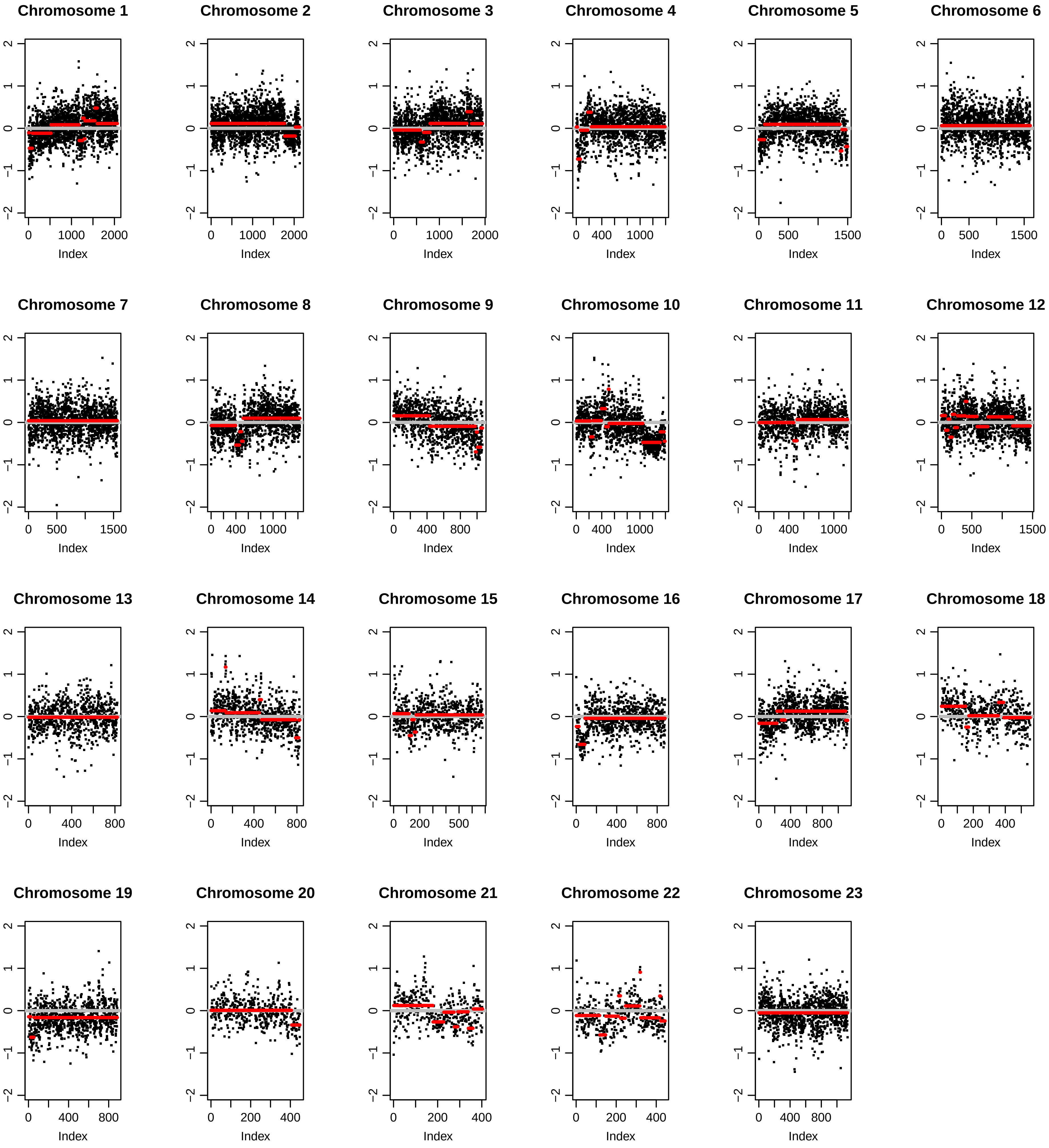

Supplemental Figure 2A (Patient 1: Liver)

LIB.021181pc3.1

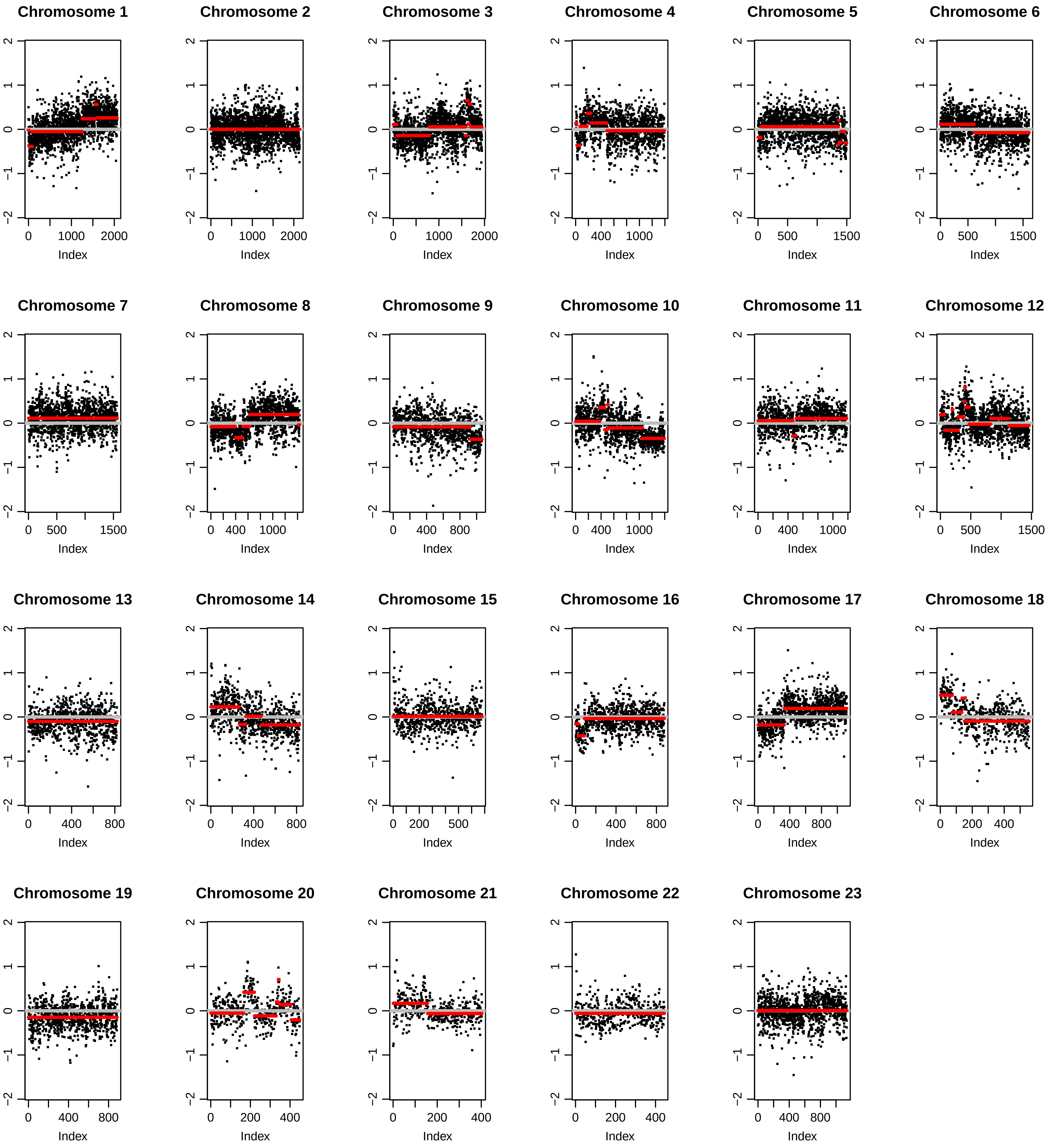

Supplemental Figure 2B (Patient 3: Right Lower Lung) **LIB.021182pc3.1**

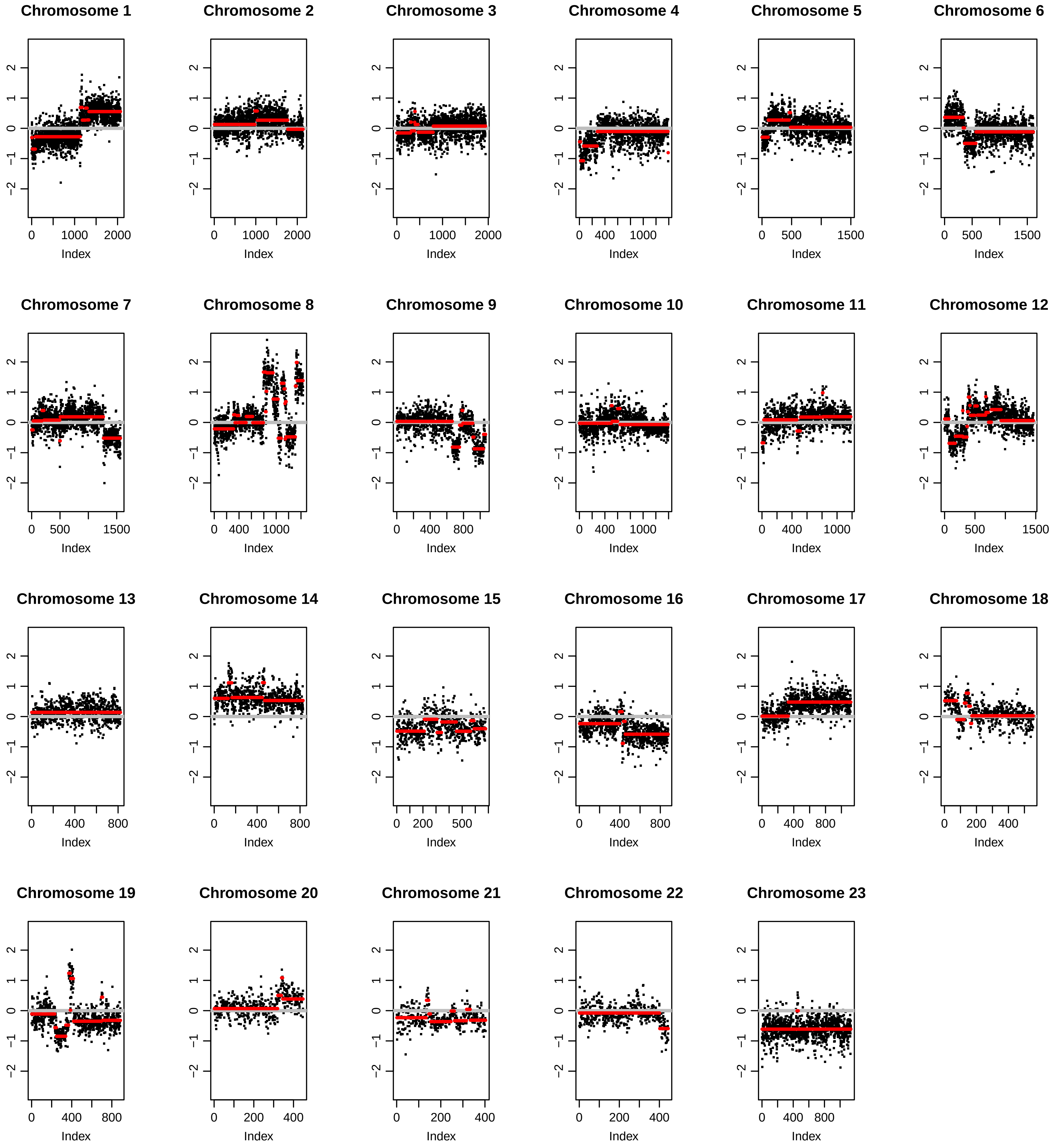

Supplemental Figure 2B (Patient 3: Right Upper Lung) **LIB.021188pc3.1**

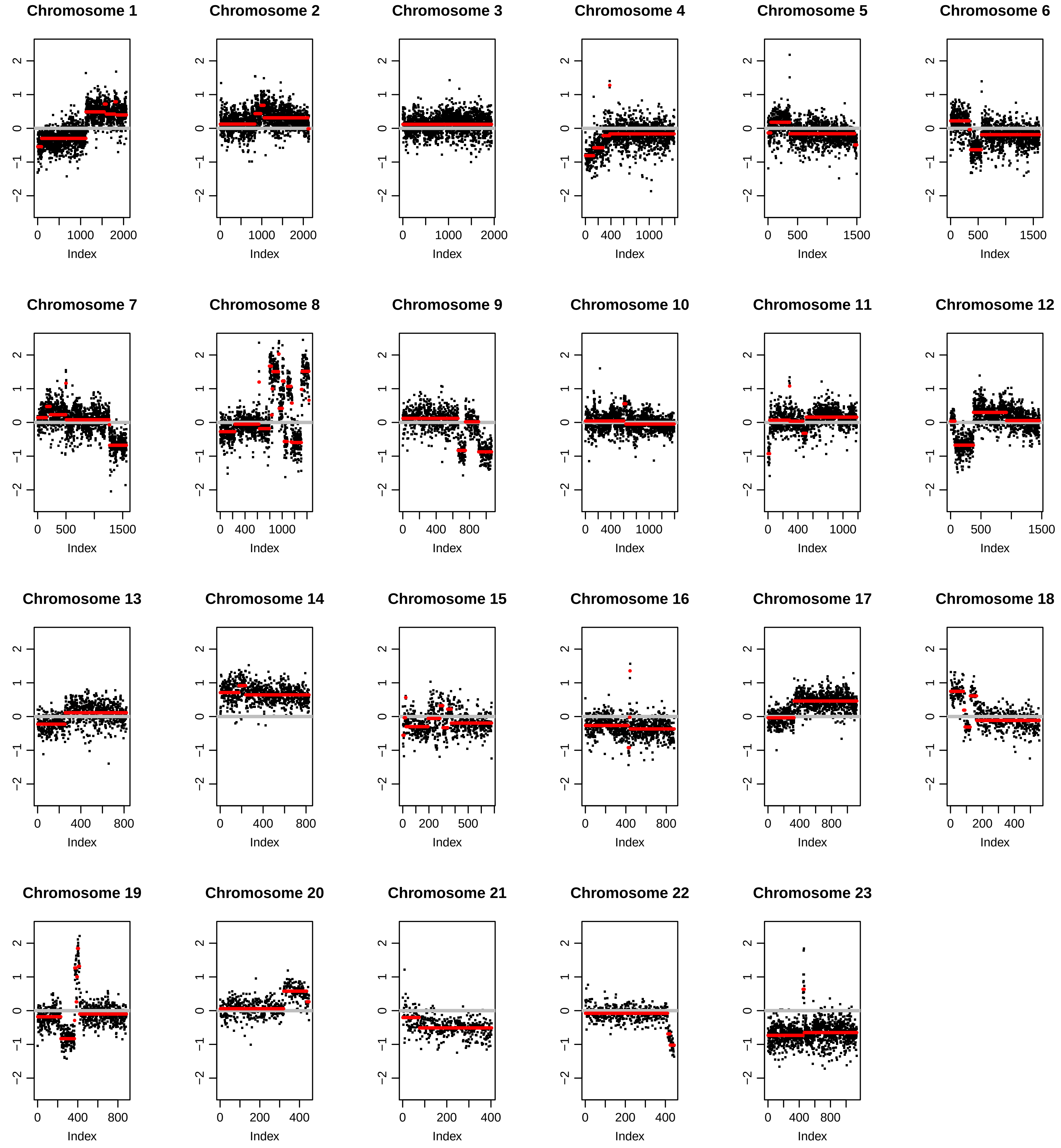

Supplemental Figure 2C (Patient 4: Right Lung) **LIB.021177pc3.1**

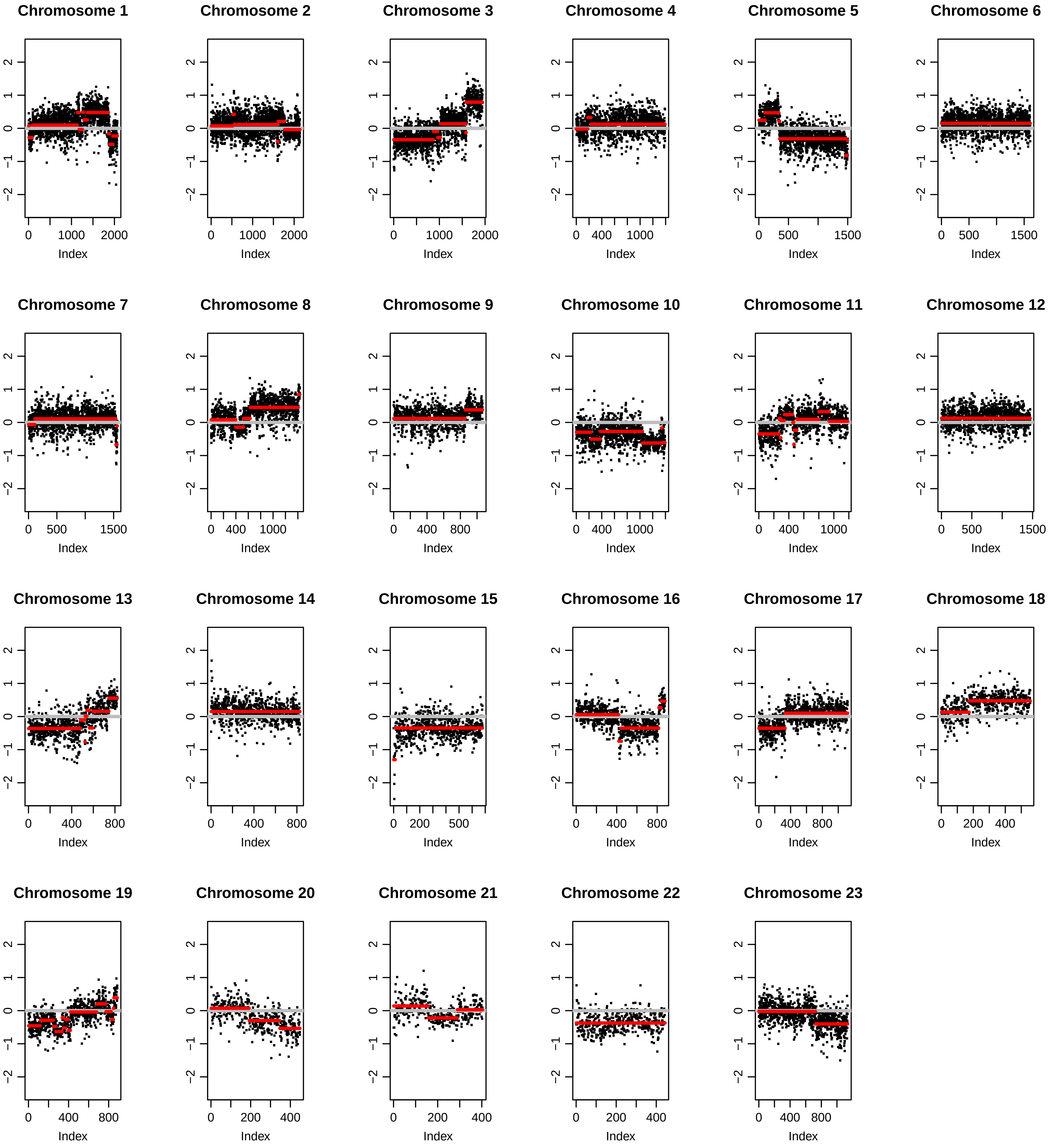

Supplemental Figure 2C (Patient 4: Lymph Node)

LIB.021191pc3.1

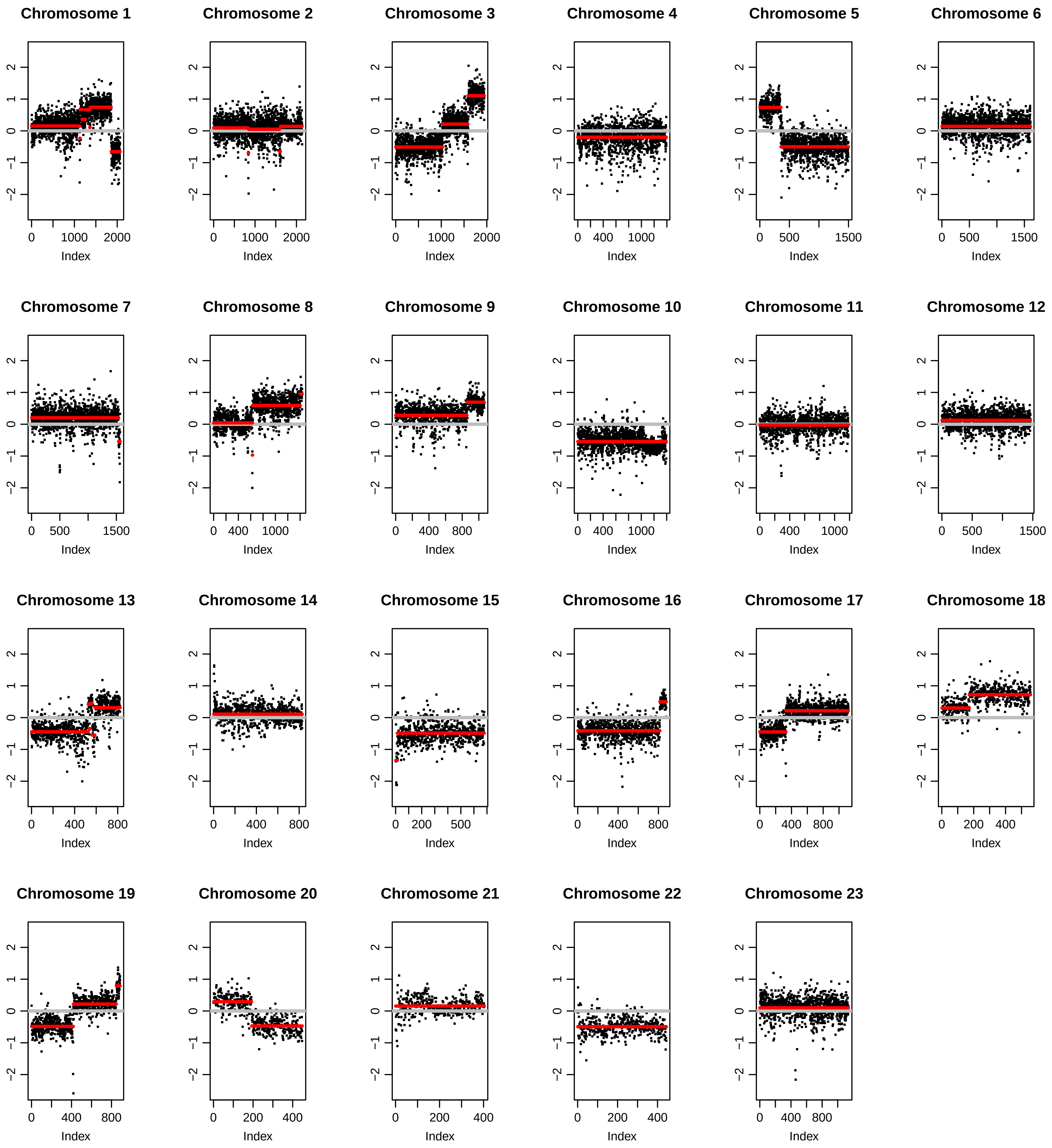

Supplemental Figure 2C (Patient 4: Liver)

LIB.021186pc3.1

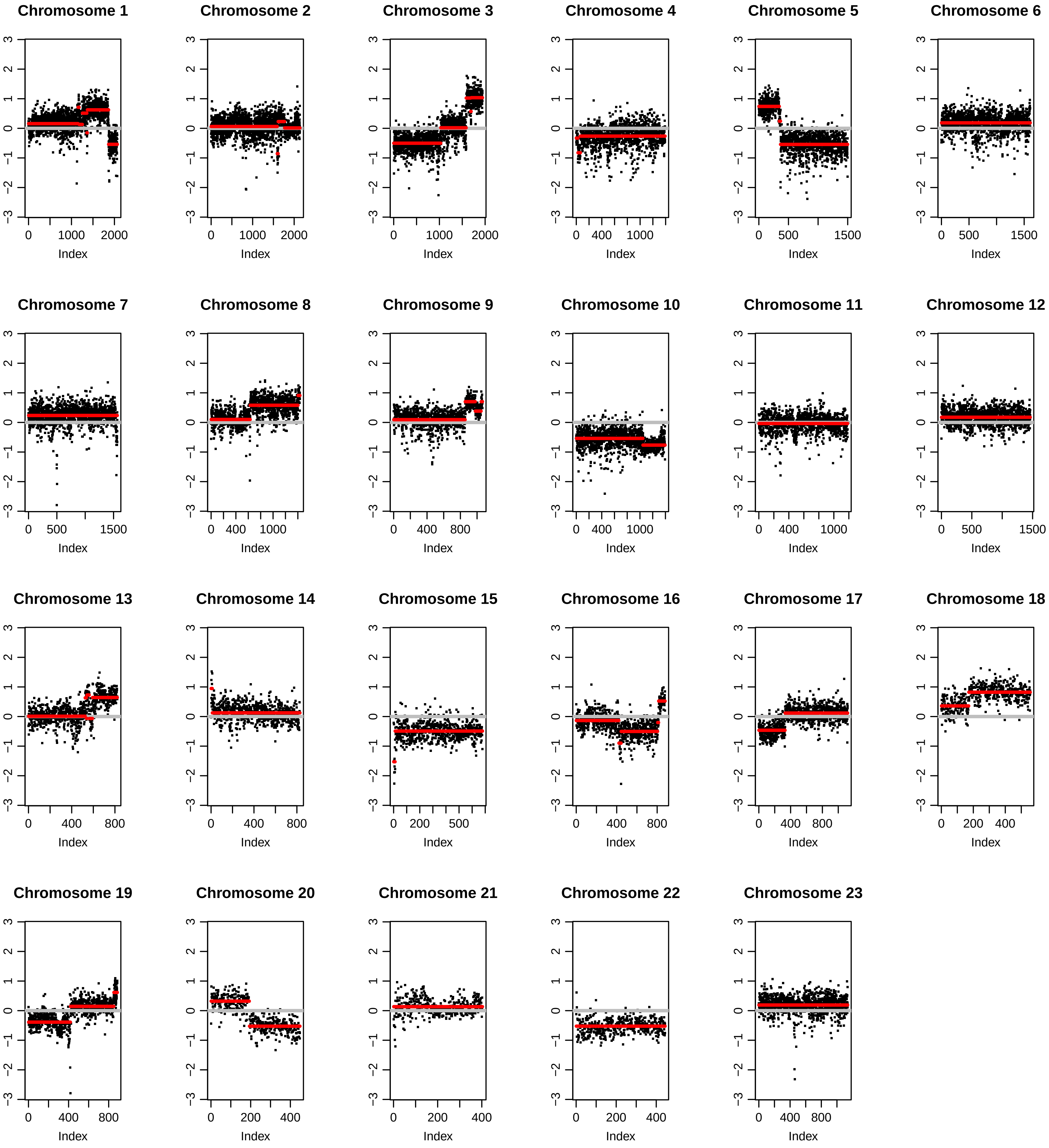

Supplemental Figure 2D (Patient 6: Left lower lung)

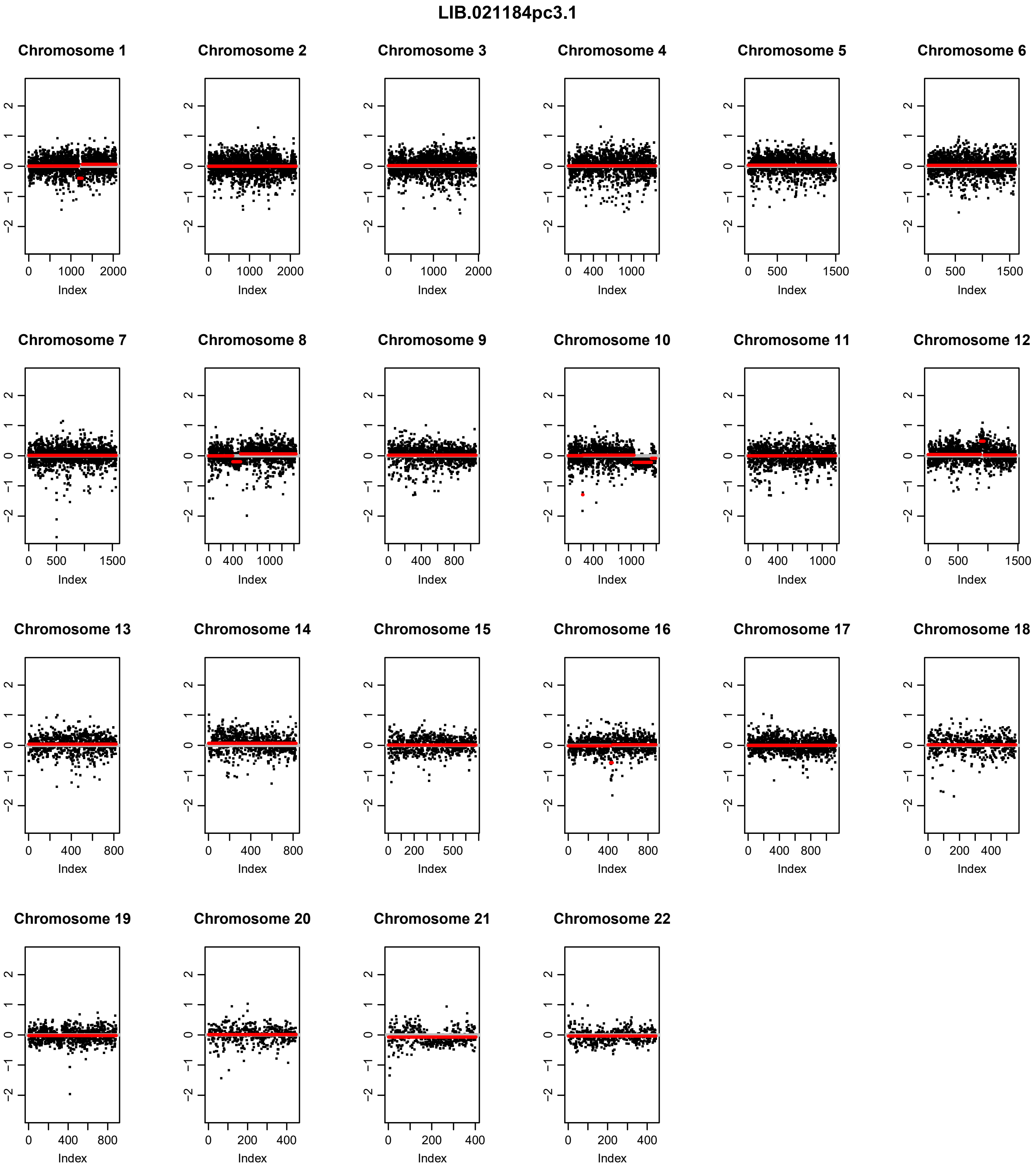

Supplemental Figure 2D (Patient 6: Lymph Node)

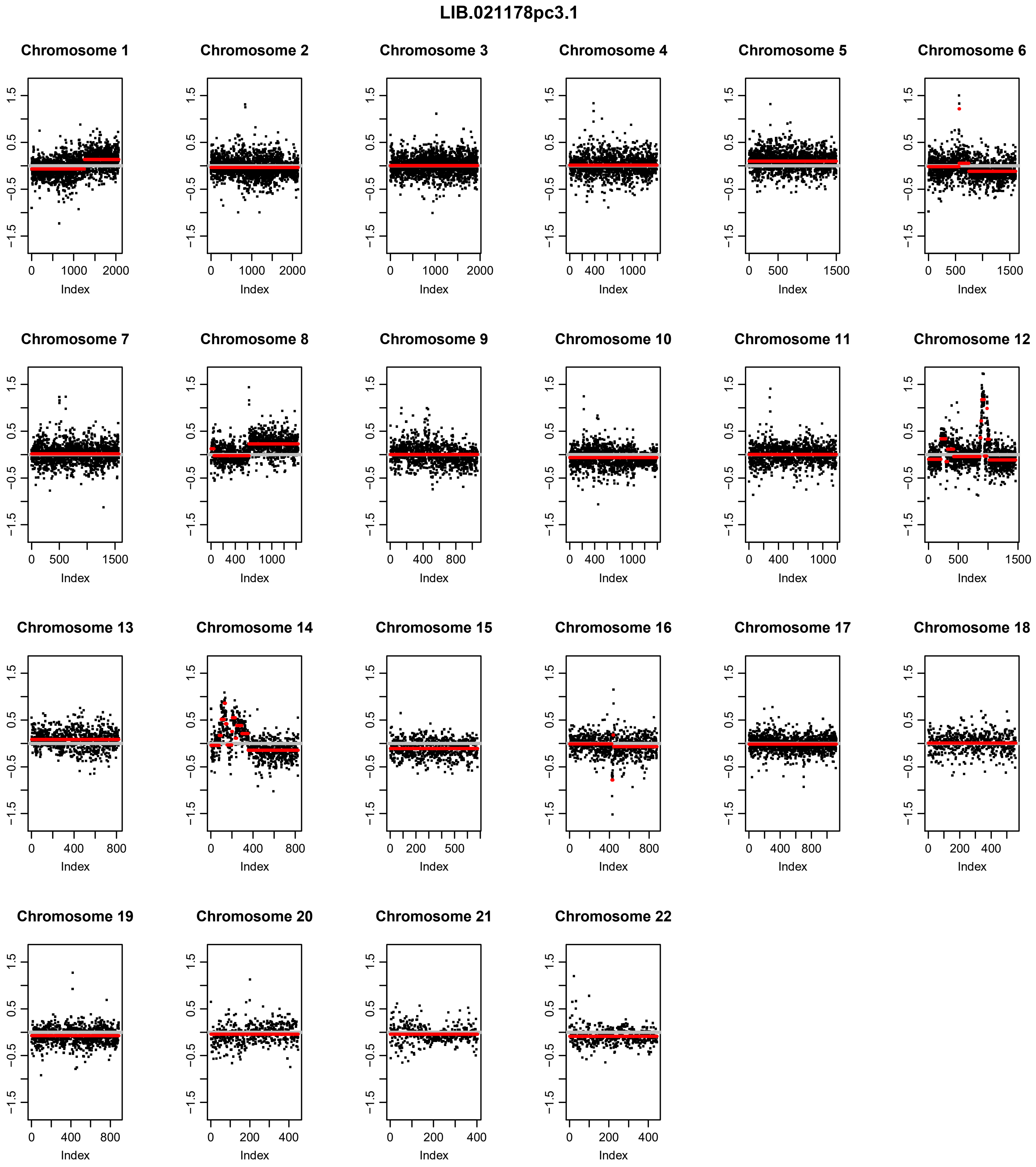

Supplemental Figure 2E (Patient 7: Left Hilar Lung) **LIB.021190pc3.1**

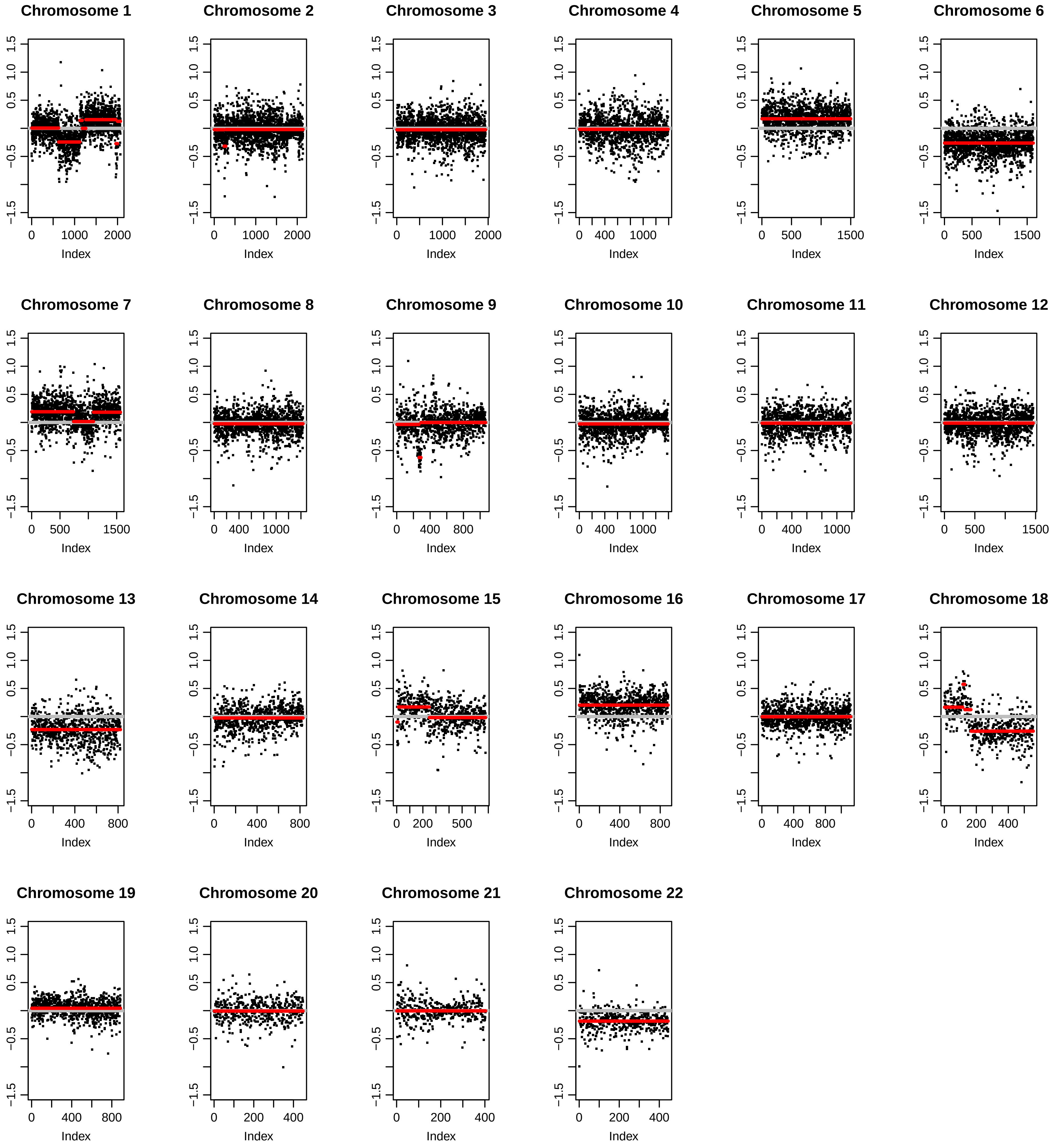

Supplemental Figure 2E (Patient 7: Lymph Node)

LIB.021189pc3.1

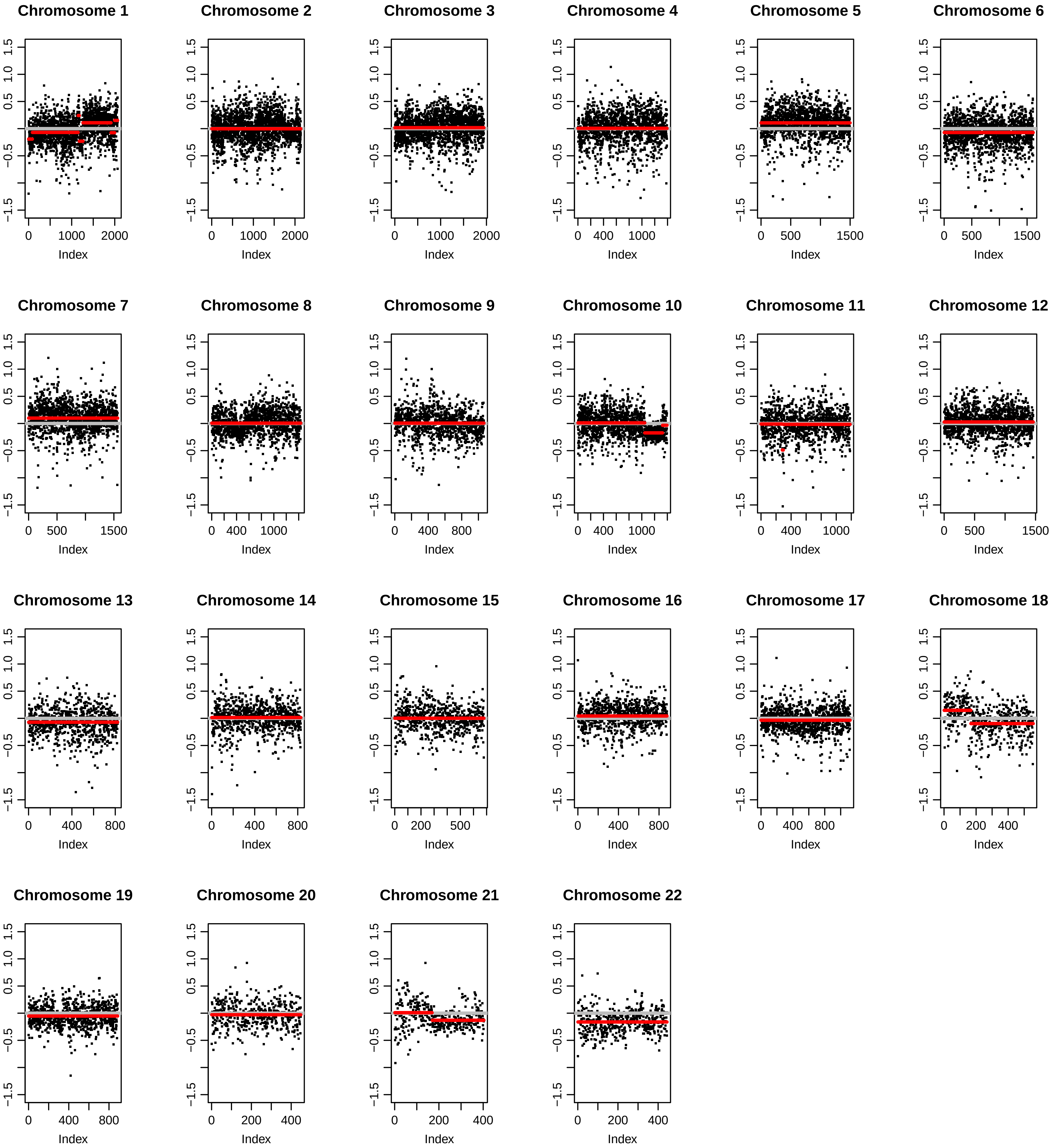

Supplemental Figure 3

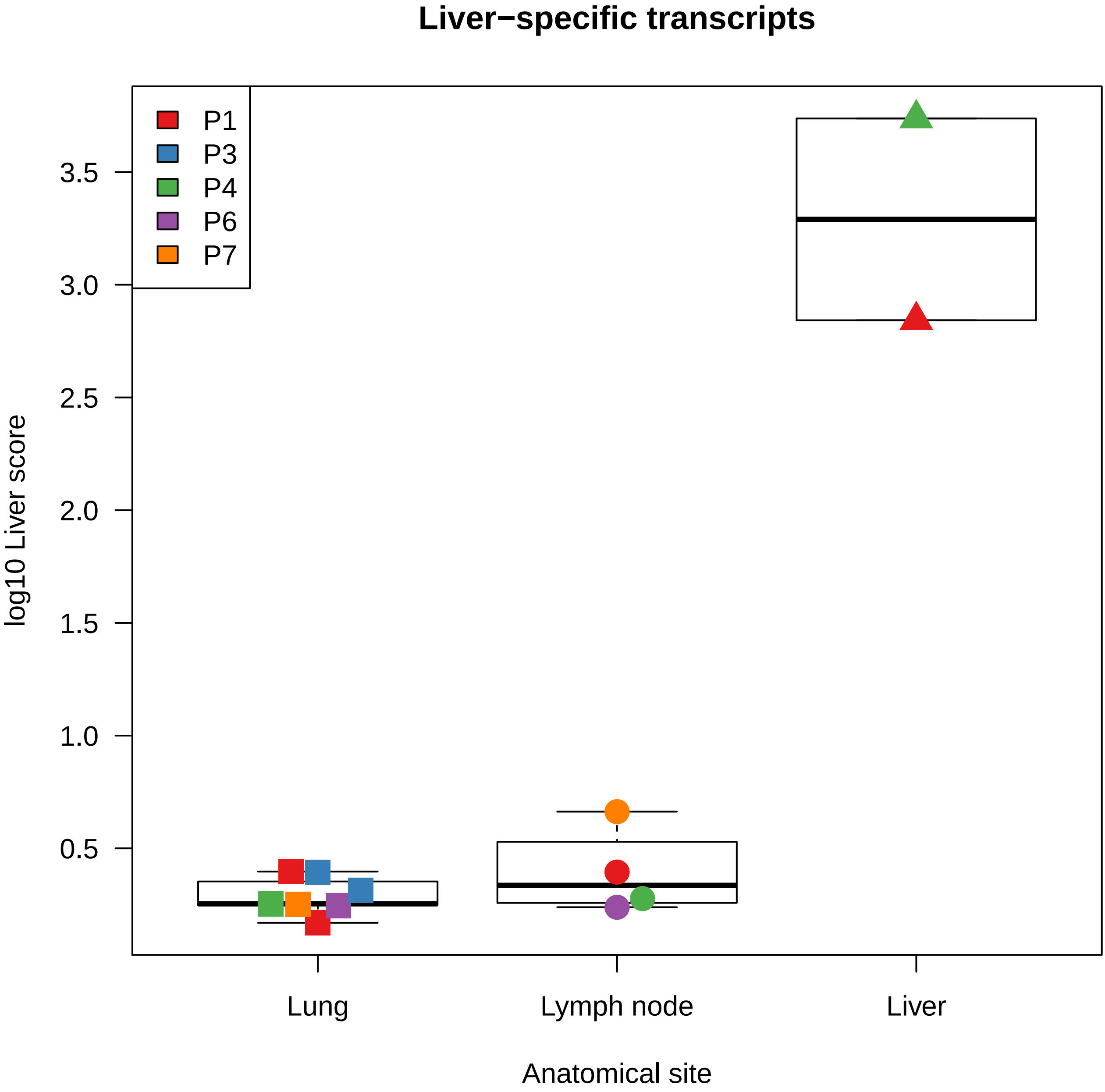

Supplement: Supplementary file 1 [file CAM4-9-225-s001.pdf]
