# Supplementary material for: A community‐based lung cancer rapid tissue donation protocol provides high‐quality drug‐resistant specimens for proteogenomic analyses
Source: Cancer Med. 2019 Nov 20;9(1):225–37. doi: 10.1002/cam4.2670 (PMC6943158; doi:10.1002/cam4.2670)
Supplement: Supplementary file 2 [file CAM4-9-225-s002.pdf]

Supplementary Table 1: Applied cutoffs for IHC markers based on semi-quantitative pathological assessment

Ki67 (%TPS)	Low: 0-25	Medium: 25-50	High: over 50
CD8 (%)	Low: below 1	Medium: 1-5	High: over 5
PD-L1 (%TPS)	Negative: below 1	Positive: ≥1	

Supplementary Table 2: Coding Regions

Gene Symbol	Gene Description	Entrez Gene ID
ABL1	c-abl oncogene 1, receptor tyrosine kinase	25
ABL2	v-abl Abelson murine leukemia viral oncogene homolog 2 (arg, Abelson-related gene)	27
ACTL6A	actin-like 6A	86
ACTL6B	actin-like 6B	51412
ACVR1B	activin A receptor, type IB	91
ACVR2A	activin A receptor type 2A	92
ADGRA2	adhesion G protein-coupled receptor A2	25960
AFF2	AF4/FMR2 family, member 2	2334
AJUBA	ajuba LIM protein	84962
AKT1	v-akt murine thymoma viral oncogene homolog 1	207
AKT2	v-akt murine thymoma viral oncogene homolog 2	208
AKT3	v-akt murine thymoma viral oncogene homolog 3 (protein kinase B, gamma)	10000
ALK	anaplastic lymphoma receptor tyrosine kinase	238
ALOX12B	arachidonate 12-lipoxygenase, 12R type	242
AMER1	APC membrane recruitment protein 1	139285
ANAPC1	anaphase promoting complex subunit 1	64682
ANO1	anoctamin 1, calcium activated chloride channel	55107
APC	adenomatous polyposis coli	324
AR	androgen receptor	367
ARAF	v-raf murine sarcoma 3611 viral oncogene homolog	369
ARFRP1	ADP-ribosylation factor related protein 1	10139
ARHGEF39	Rho guanine nucleotide exchange factor (GEF) 39	84904
ARID1A	AT rich interactive domain 1A (SWI-like)	8289
ARID1B	AT rich interactive domain 1B (SWI1-like)	57492
ARID2	AT rich interactive domain 2 (ARID, RFX-like)	196528
ARID5B	AT-rich interaction domain 5B	84159
ATAD2	ATPase family, AAA domain containing 2	29028

ATM	similar to Serine-protein kinase ATM (Ataxia telangiectasia mutated) (A-T, mutated); ataxia telangiectasia mutated	472
ATR	ataxia telangiectasia and Rad3 related; similar to ataxia telangiectasia and Rad3 related protein	545
ATRX	alpha thalassemia/mental retardation syndrome X-linked (RAD54 homolog, S. cerevisiae)	546
AURKA	aurora kinase A; aurora kinase A pseudogene 1	6790
AURKB	aurora kinase B	9212
AXIN1	axin 1	8312
AXL	AXL receptor tyrosine kinase	558
B3GAT1	beta-1,3-glucuronyltransferase 1 (glucuronosyltransferase P)	27087
BACH1	BTB and CNC homology 1, basic leucine zipper transcription factor 1	571
BACH2	BTB and CNC homology 1, basic leucine zipper transcription factor 2	60468
BAP1	BRCA1 associated protein-1 (ubiquitin carboxy-terminal hydrolase)	8314
BARD1	BRCA1 associated RING domain 1	580
BCL11A	B-cell CLL/lymphoma 11A	53335
BCL11B	B-cell CLL/lymphoma 11B	64919
BCL2	B-cell CLL/lymphoma 2	596
BCL2A1	BCL2-related protein A1	597
BCL2L1	BCL2-like 1	598
BCL2L11	BCL2-like 11 (apoptosis facilitator)	10018
BCL3	B-cell CLL/lymphoma 3	602
BCL6	B-cell CLL/lymphoma 6	604
BCL7A	BCL tumor suppressor 7A	605
BCL7B	BCL tumor suppressor 7B	9275
BCL7C	BCL tumor suppressor 7C	9274
BCOR	BCL6 co-repressor	54880
BDH1	3-hydroxybutyrate dehydrogenase, type 1	622
BIRC2	baculoviral IAP repeat-containing 2	329
BIRC3	baculoviral IAP repeat-containing 3	330
BIRC7	baculoviral IAP repeat-containing 7	79444
BLM	Bloom syndrome, RecQ helicase-like	641
BOK	BCL2-related ovarian killer	666

BRAF	v-raf murine sarcoma viral oncogene homolog B1	673
BRCA1	breast cancer 1, early onset	672
BRCA2	breast cancer 2, early onset	675
BRD4	bromodomain containing 4	23476
BRD7	bromodomain containing 7; bromodomain containing 7 pseudogene 2	29117
BRD9	bromodomain containing 9	65980
BRIP1	BRCA1 interacting protein C-terminal helicase 1	83990
BTG1	B-cell translocation gene 1, anti-proliferative	694
BTK	Bruton agammaglobulinemia tyrosine kinase	695
CABLES1	Cdk5 and Abl enzyme substrate 1	91768
CARD11	caspase recruitment domain family, member 11	84433
CASP8	caspase 8, apoptosis-related cysteine peptidase	841
CBFB	core-binding factor, beta subunit	865
CBL	Cas-Br-M (murine) ecotropic retroviral transforming sequence	867
CCDC89	coiled-coil domain containing 89	220388
CCND1	cyclin D1	595
CCND2	cyclin D2	894
CCND3	cyclin D3	896
CCNE1	cyclin E1	898
CCSER1	coiled-coil serine rich protein 1	401145
CD19	CD19 molecule	930
CD274	CD274 molecule	29126
CD79A	CD79a molecule, immunoglobulin-associated alpha	973
CD79B	CD79b molecule, immunoglobulin-associated beta	974
CDC73	cell division cycle 73, Paf1/RNA polymerase II complex component, homolog (S. cerevisiae)	79577
CDH1	cadherin 1, type 1, E-cadherin (epithelial)	999
CDH13	cadherin 13, H-cadherin (heart)	1012
CDH20	cadherin 20, type 2	28316
CDH3	cadherin 3	1001

CDH5	cadherin 5, type 2 (vascular endothelium)	1003
CDH6	cadherin 6	1004
CDK12	Cdc2-related kinase, arginine/serine-rich	51755
CDK17	PCTAIRE protein kinase 2	5128
CDK2	cyclin dependent kinase 2	1017
CDK4	cyclin-dependent kinase 4	1019
CDK6	cyclin-dependent kinase 6	1021
CDKN1A	cyclin dependent kinase inhibitor 1A	1026
CDKN1B	cyclin-dependent kinase inhibitor 1B (p27, Kip1)	1027
CDKN2A	cyclin-dependent kinase inhibitor 2A (melanoma, p16, inhibits CDK4)	1029
CDKN2B	cyclin-dependent kinase inhibitor 2B (p15, inhibits CDK4)	1030
CDKN2C	cyclin-dependent kinase inhibitor 2C (p18, inhibits CDK4)	1031
CEBPA	CCAAT/enhancer binding protein (C/EBP), alpha	1050
CHD1	chromodomain helicase DNA binding protein 1	1105
CHD3	chromodomain helicase DNA binding protein 3	1107
CHD4	chromodomain helicase DNA binding protein 4	1108
CHD5	chromodomain helicase DNA binding protein 5	26038
CHEK2	protein kinase CHK2-like; CHK2 checkpoint homolog (S. pombe); similar to hCG1983233	11200
CHFR	checkpoint with forkhead and ring finger domains	55743
CHSY3	chondroitin sulfate synthase 3	337876
CHUK	conserved helix-loop-helix ubiquitous kinase	1147
CIC	capicua homolog (Drosophila)	23152
COPS5	COP9 constitutive photomorphogenic homolog subunit 5 (Arabidopsis)	10987
COX18	COX18 cytochrome c oxidase assembly homolog (S. cerevisiae)	285521
CPSF3	cleavage and polyadenylation specific factor 3	51692
CREBBP	CREB binding protein	1387

CRKL	v-crk sarcoma virus CT10 oncogene homolog (avian)-like	1399
CSF1	colony stimulating factor 1	1435
CSF1R	colony stimulating factor 1 receptor	1436
CTCF	CCCTC-binding factor (zinc finger protein)	10664
CTLA4	cytotoxic T-lymphocyte-associated protein 4	1493
CTNNB1	catenin (cadherin-associated protein), beta 1, 88kDa	1499
CUL1	cullin 1	8454
CUL4A	cullin 4A	8451
CUL4B	cullin 4B	8450
CUX1	cut like homeobox 1	1523
DAXX	death-domain associated protein	1616
DCC	deleted in colorectal carcinoma	1630
DDR2	discoidin domain receptor tyrosine kinase 2	4921
DDX11	DEAD/H (Asp-Glu-Ala-Asp/His) box polypeptide 11 (CHL1-like helicase homolog, S. cerevisiae)	1663
DEPDC5	DEP domain containing 5	9681
DICER1	dicer 1, ribonuclease type III	23405
DIS3	DIS3 mitotic control homolog (S. cerevisiae)	22894
DKK1	dickkopf homolog 1 (Xenopus laevis)	22943
DLGAP2	discs, large (Drosophila) homolog-associated protein 2	9228
DNMT3A	DNA (cytosine-5-)-methyltransferase 3 alpha	1788
DOT1L	DOT1-like, histone H3 methyltransferase (S. cerevisiae)	84444
DPF1	D4, zinc and double PHD fingers family 1	8193
DPF2	D4, zinc and double PHD fingers family 2	5977
DPF3	D4, zinc and double PHD fingers, family 3	8110
DSG3	desmoglein 3 (pemphigus vulgaris antigen)	1830
DTX2	deltex homolog 2 (Drosophila)	113878
DUSP4	dual specificity phosphatase 4	1846
EED	embryonic ectoderm development	8726
EGFR	epidermal growth factor receptor (erythroblastic leukemia viral (v-erb-b) oncogene homolog, avian)	1956

EIF1AX	eukaryotic translation initiation factor 1A, X-linked	1964
ELK3	ELK3, ETS-domain protein (SRF accessory protein 2)	2004
ELMO1	engulfment and cell motility 1	9844
EML4	echinoderm microtubule associated protein like 4	27436
EMSY	EMSY transcriptional repressor, BRCA2 interacting	56946
EP300	E1A binding protein p300	2033
EPAS1	endothelial PAS domain protein 1	2034
EPHA2	EPH receptor A2	1969
EPHA3	EPH receptor A3	2042
EPHA6	EPH receptor A6	285220
EPHA7	EPH receptor A7	2045
ERBB2	v-erb-b2 erythroblastic leukemia viral oncogene homolog 2, neuro/glioblastoma derived oncogene homolog (avian)	2064
ERBB3	v-erb-b2 erythroblastic leukemia viral oncogene homolog 3 (avian)	2065
ERBB4	v-erb-a erythroblastic leukemia viral oncogene homolog 4 (avian)	2066
ERG	v-ets erythroblastosis virus E26 oncogene homolog (avian)	2078
ESR1	estrogen receptor 1	2099
ESR2	estrogen receptor 2 (ER beta)	2100
ETV1	ets variant 1	2115
ETV4	ets variant 4	2118
ETV5	ets variant 5	2119
ETV6	ets variant 6	2120
EWSR1	similar to Ewing sarcoma breakpoint region 1; Ewing sarcoma breakpoint region 1	2130
EZH2	enhancer of zeste homolog 2 (Drosophila)	2146
FANCA	Fanconi anemia, complementation group A	2175
FANCC	Fanconi anemia, complementation group C	2176
FANCD2	Fanconi anemia, complementation group D2	2177
FANCE	Fanconi anemia, complementation group E	2178
FANCF	Fanconi anemia, complementation group F	2188
FANCG	Fanconi anemia, complementation group G	2189

FANCI	Fanconi anemia, complementation group I	55215
FANCL	Fanconi anemia, complementation group L	55120
FANCM	Fanconi anemia, complementation group M	57697
FAT1	FAT tumor suppressor homolog 1 (Drosophila)	2195
FAT3	FAT tumor suppressor homolog 3 (Drosophila)	120114
FBXW7	F-box and WD repeat domain containing 7	55294
FGF10	fibroblast growth factor 10	2255
FGF12	fibroblast growth factor 12	2257
FGF14	fibroblast growth factor 14	2259
FGF19	fibroblast growth factor 19	9965
FGF23	fibroblast growth factor 23	8074
FGF3	fibroblast growth factor 3 (murine mammary tumor virus integration site (v-int-2) oncogene homolog)	2248
FGF4	fibroblast growth factor 4	2249
FGF6	fibroblast growth factor 6	2251
FGF7	fibroblast growth factor 7	2252
FGFR1	fibroblast growth factor receptor 1	2260
FGFR2	fibroblast growth factor receptor 2	2263
FGFR3	fibroblast growth factor receptor 3	2261
FGFR4	fibroblast growth factor receptor 4	2264
FH	fumarate hydratase	2271
FLG	filaggrin	2312
FLG2	filaggrin family member 2	388698
FLI1	Friend leukemia virus integration 1	2313
FLRT3	fibronectin leucine rich transmembrane protein 3	23767
FLT1	fms-related tyrosine kinase 1 (vascular endothelial growth factor/vascular permeability factor receptor)	2321
FLT3	fms-related tyrosine kinase 3	2322
FLT4	fms-related tyrosine kinase 4	2324
FOXA1	forkhead box A1	3169
FOXC1	forkhead box C1	2296
FOXL2	forkhead box L2	668
FOXM1	forkhead box M1	2305
FOXP1	forkhead box P1	27086
FRK	fyn related Src family tyrosine kinase	2444

FRS2	fibroblast growth factor receptor substrate 2	10818
GAB2	GRB2-associated binding protein 2	9846
GATA1	GATA binding protein 1 (globin transcription factor 1)	2623
GATA3	GATA binding protein 3	2625
GLI1	GLI family zinc finger 1	2735
GMDS	GDP-mannose 4,6-dehydratase	2762
GNA11	guanine nucleotide binding protein (G protein), alpha 11 (Gq class)	2767
GNAQ	guanine nucleotide binding protein (G protein), q polypeptide	2776
GNAS	GNAS complex locus	2778
GPC6	glypican 6	10082
GPS2	G protein pathway suppressor 2	2874
GRB2	growth factor receptor-bound protein 2	2885
GRIN2A	glutamate receptor, ionotropic, N-methyl D-aspartate 2A	2903
GSK3B	glycogen synthase kinase 3 beta	2932
GSTM1	glutathione S-transferase mu 1	2944
GSTT1	glutathione S-transferase theta 1	2952
H3F3A	H3 histone, family 3B (H3.3B); H3 histone, family 3A pseudogene; H3 histone, family 3A; similar to H3 histone, family 3B; similar to histone H3.3B	3020
HAVCR2	hepatitis A virus cellular receptor 2	84868
HAX1	HCLS1 associated protein X-1	10456
HDAC10	histone deacetylase 10	83933
HGF	hepatocyte growth factor (hepapoietin A; scatter factor)	3082
HIF1A	hypoxia inducible factor 1 alpha subunit	3091
HIST1H3B	histone cluster 1, H3b	8358
HLA-A	major histocompatibility complex, class I, A	3105
HLA-B	major histocompatibility complex, class I, B	3106
HLA-C	major histocompatibility complex, class I, C	3107
HNF4A	hepatocyte nuclear factor 4 alpha	3172
HOXA13	homeobox A13	3209
HRAS	v-Ha-ras Harvey rat sarcoma viral oncogene homolog	3265
HRNR	hornerin	388697

HSP90AA1	heat shock protein 90kDa alpha (cytosolic), class A member 2; heat shock protein 90kDa alpha (cytosolic), class A member 1	3320
HSP90AB1	heat shock protein 90kDa alpha (cytosolic), class B member 1	3326
HSP90B1	heat shock protein 90kDa beta (Grp94), member 1	7184
IDH1	isocitrate dehydrogenase 1 (NADP+), soluble	3417
IDH2	isocitrate dehydrogenase 2 (NADP+), mitochondrial	3418
IGF1	insulin-like growth factor 1 (somatomedin C)	3479
IGF1R	insulin-like growth factor 1 receptor	3480
IGF2	insulin-like growth factor 2 (somatomedin A); insulin; INS-IGF2 readthrough transcript	3481
IGF2R	insulin-like growth factor 2 receptor	3482
IKBKE	inhibitor of kappa light polypeptide gene enhancer in B-cells, kinase epsilon	9641
IL7R	interleukin 7 receptor	3575
ING1	inhibitor of growth family, member 1	3621
INPP4B	inositol polyphosphate-4-phosphatase type II B	8821
INSR	insulin receptor	3643
INTS4	integrator complex subunit 4	92105
IRAK1	interleukin-1 receptor-associated kinase 1	3654
IRF2	interferon regulatory factor 2	3660
IRF2BP1	interferon regulatory factor 2 binding protein 1	26145
IRF4	interferon regulatory factor 4	3662
IRS2	insulin receptor substrate 2	8660
JAK1	Janus kinase 1	3716
JAK2	Janus kinase 2	3717
JAK3	Janus kinase 3	3718
JUN	jun proto-oncogene	3725
KAT6A	K(lysine) acetyltransferase 6A	7994
KAT6B	K(lysine) acetyltransferase 6B	23522
KDM1A	lysine (K)-specific demethylase 1	23028
KDM2A	lysine (K)-specific demethylase 2A	22992
KDM2B	lysine (K)-specific demethylase 2B	84678

KDM3B	lysine (K)-specific demethylase 3B	51780
KDM4A	lysine (K)-specific demethylase 4A	9682
KDM4B	lysine (K)-specific demethylase 4B	23030
KDM4C	lysine (K)-specific demethylase 4C	23081
KDM5A	lysine (K)-specific demethylase 5A	5927
KDM5C	lysine (K)-specific demethylase 5C	8242
KDM6A	lysine (K)-specific demethylase 6A	7403
KDR	kinase insert domain receptor (a type III receptor tyrosine kinase)	3791
KEAP1	kelch-like ECH-associated protein 1	9817
KIF5B	kinesin family member 5B	3799
KIT	similar to Mast/stem cell growth factor receptor precursor (SCFR) (Proto-oncogene tyrosine-protein kinase Kit) (c-kit) (CD117 antigen); v-kit Hardy-Zuckerman 4 feline sarcoma viral oncogene homolog	3815
KLF4	Kruppel-like factor 4 (gut)	9314
KLHL6	kelch-like 6 (Drosophila)	89857
KMT2A	myeloid/lymphoid or mixed-lineage leukemia (trithorax homolog, Drosophila)	4297
KMT2B	myeloid/lymphoid or mixed-lineage leukemia 4	9757
KMT2C	myeloid/lymphoid or mixed-lineage leukemia 3	58508
KMT2D	myeloid/lymphoid or mixed-lineage leukemia 2	8085
KRAS	v-Ki-ras2 Kirsten rat sarcoma viral oncogene homolog	3845
KYNU	kynureninase (L-kynurenine hydrolase)	8942
LAG3	lymphocyte activating 3	3902
LATS1	LATS, large tumor suppressor, homolog 1 (Drosophila)	9113
LATS2	LATS, large tumor suppressor, homolog 2 (Drosophila)	26524
LMO1	LIM domain only 1 (rhombotin 1)	4004
LONRF1	LON peptidase N-terminal domain and ring finger 1	91694
LTK	leukocyte receptor tyrosine kinase	4058
MACROD2	MACRO domain containing 2	140733
MAP2K1	mitogen-activated protein kinase kinase 1	5604

MAP2K2	mitogen-activated protein kinase kinase 2 pseudogene; mitogen-activated protein kinase kinase 2	5605
MAP2K4	mitogen-activated protein kinase kinase 4	6416
MAP3K1	mitogen-activated protein kinase kinase kinase 1	4214
MAP3K13	mitogen-activated protein kinase kinase kinase 13	9175
MAP3K7	mitogen-activated protein kinase kinase kinase 7	6885
MAPK1	mitogen-activated protein kinase 1	5594
MAPK3	hypothetical LOC100271831; mitogen-activated protein kinase 3	5595
MAPK7	mitogen-activated protein kinase 7	5598
MAPKBP1	mitogen-activated protein kinase binding protein 1	23005
MAX	MYC associated factor X	4149
MCL1	myeloid cell leukemia sequence 1 (BCL2-related)	4170
MDM2	Mdm2 p53 binding protein homolog (mouse)	4193
MDM4	Mdm4 p53 binding protein homolog (mouse)	4194
MECOM	ecotropic viral integration site 1	2122
MED12	mediator complex subunit 12	9968
MELK	maternal embryonic leucine zipper kinase	9833
MEN1	multiple endocrine neoplasia I	4221
MET	met proto-oncogene (hepatocyte growth factor receptor)	4233
MGMT	O-6-methylguanine-DNA methyltransferase	4255
MITF	microphthalmia-associated transcription factor	4286
MLH1	mutL homolog 1, colon cancer, nonpolyposis type 2 (E. coli)	4292
MLH3	mutL homolog 3 (E. coli)	27030
MOB1A	MOB kinase activator 1A	55233
MOB1B	MOB kinase activator 1B	92597
MORC1	MORC family CW-type zinc finger 1	27136
MPL	myeloproliferative leukemia virus oncogene	4352
MSH2	mutS homolog 2, colon cancer, nonpolyposis type 1 (E. coli)	4436

MSH3	mutS homolog 3 (E. coli)	4437
MSH6	mutS homolog 6 (E. coli)	2956
MST1	macrophage stimulating 1 (hepatocyte growth factor-like)	4485
MST1R	macrophage stimulating 1 receptor (c-met-related tyrosine kinase)	4486
MTAP	methylthioadenosine phosphorylase	4507
MTOR	mechanistic target of rapamycin (serine/threonine kinase)	2475
MYB	v-myb myeloblastosis viral oncogene homolog (avian)	4602
MYC	v-myc myelocytomatosis viral oncogene homolog (avian)	4609
MYCL	MYCL proto-oncogene, bHLH transcription factor	4610
MYCN	v-myc myelocytomatosis viral related oncogene, neuroblastoma derived (avian)	4613
MYH9	myosin, heavy chain 9, non-muscle	4627
NAALADL2	N-acetylated alpha-linked acidic dipeptidase-like 2	254827
NCOR1	nuclear receptor co-repressor 1	9611
NEGR1	neuronal growth regulator 1	257194
NF1	neurofibromin 1	4763
NF2	neurofibromin 2 (merlin)	4771
NFE2L2	nuclear factor (erythroid-derived 2)-like 2	4780
NFKBIA	nuclear factor of kappa light polypeptide gene enhancer in B-cells inhibitor, alpha	4792
NKX2-1	NK2 homeobox 1	7080
NOTCH1	Notch homolog 1, translocation-associated (Drosophila)	4851
NOTCH2	Notch homolog 2 (Drosophila)	4853
NOTCH3	Notch homolog 3 (Drosophila)	4854
NOTCH4	Notch homolog 4 (Drosophila)	4855
NPM1	nucleophosmin 1 (nucleolar phosphoprotein B23, numatrin) pseudogene 21; hypothetical LOC100131044; similar to nucleophosmin 1; nucleophosmin (nucleolar phosphoprotein B23, numatrin)	4869
NRAS	neuroblastoma RAS viral (v-ras) oncogene homolog	4893

NSD1	nuclear receptor binding SET domain protein 1	64324
NT5C2	5'-nucleotidase, cytosolic II	22978
NTNG1	netrin G1	22854
NTRK1	neurotrophic tyrosine kinase, receptor, type 1	4914
NTRK2	neurotrophic tyrosine kinase, receptor, type 2	4915
NTRK3	neurotrophic tyrosine kinase, receptor, type 3	4916
PAF1	Paf1, RNA polymerase II associated factor, homolog (S. cerevisiae)	54623
PAK7	p21 protein (Cdc42/Rac)-activated kinase 7	57144
PALB2	partner and localizer of BRCA2	79728
PARD6G	par-6 partitioning defective 6 homolog gamma (C. elegans)	84552
PARK2	Parkinson disease (autosomal recessive, juvenile) 2, parkin	5071
PARP1	poly (ADP-ribose) polymerase 1	142
PARP10	poly (ADP-ribose) polymerase family, member 10	84875
PARP2	poly (ADP-ribose) polymerase 2	10038
PARP3	poly (ADP-ribose) polymerase family, member 3	10039
PARP4	poly (ADP-ribose) polymerase family, member 4	143
PAX3	paired box 3	5077
PBRM1	polybromo 1	55193
PCDH15	protocadherin 15	65217
PDCD1	programmed cell death 1	5133
PDCD1LG2	programmed cell death 1 ligand 2	80380
PDE4D	phosphodiesterase 4D, cAMP-specific (phosphodiesterase E3 dunce homolog, Drosophila)	5144
PDGFRA	platelet-derived growth factor receptor, alpha polypeptide	5156
PDGFRB	platelet-derived growth factor receptor, beta polypeptide	5159
PDK1	pyruvate dehydrogenase kinase, isozyme 1	5163
PDPK1	3-phosphoinositide dependent protein kinase-1	5170

PEA15	phosphoprotein enriched in astrocytes 15	8682
PEG10	paternally expressed 10	23089
PGM5	phosphoglucomutase 5	5239
PHF10	PHD finger protein 10	55274
PHF12	PHD finger protein 12	57649
PHF6	PHD finger protein 6	84295
PHLPP2	PH domain and leucine rich repeat protein phosphatase 2	23035
PHOX2B	paired-like homeobox 2b	8929
PIK3C2G	phosphoinositide-3-kinase, class 2, gamma polypeptide	5288
PIK3C3	phosphoinositide-3-kinase, class 3	5289
PIK3CA	phosphoinositide-3-kinase, catalytic, alpha polypeptide	5290
PIK3CB	phosphoinositide-3-kinase, catalytic, beta polypeptide	5291
PIK3CG	phosphoinositide-3-kinase, catalytic, gamma polypeptide	5294
PIK3R1	phosphoinositide-3-kinase, regulatory subunit 1 (alpha)	5295
PIK3R2	phosphoinositide-3-kinase, regulatory subunit 2 (beta)	5296
PIK3R3	phosphoinositide-3-kinase, regulatory subunit 3 (gamma)	8503
PIK3R4	phosphoinositide-3-kinase, regulatory subunit 4	30849
PIK3R5	phosphoinositide-3-kinase, regulatory subunit 5	23533
PIK3R6	phosphoinositide-3-kinase, regulatory subunit 6	146850
PIM1	pim-1 oncogene	5292
PIM2	pim-2 oncogene	11040
PIM3	pim-3 oncogene	415116
PKHD1	polycystic kidney and hepatic disease 1 (autosomal recessive)	5314
PLAT	plasminogen activator, tissue	5327
PLK2	polo-like kinase 2 (Drosophila)	10769
PMS2	PMS2 postmeiotic segregation increased 2 (S. cerevisiae)	5395
POLD1	polymerase (DNA directed), delta 1, catalytic subunit 125kDa	5424

POLE	polymerase (DNA directed), epsilon	5426
PORCN	porcupine homolog (Drosophila)	64840
PPARA	peroxisome proliferator-activated receptor alpha	5465
PPM1E	protein phosphatase 1E (PP2C domain containing)	22843
PPP1R3A	protein phosphatase 1, regulatory (inhibitor) subunit 3A	5506
PPP2R1A	protein phosphatase 2 (formerly 2A), regulatory subunit A, alpha isoform	5518
PPP6C	protein phosphatase 6, catalytic subunit	5537
PRDM1	PR domain containing 1, with ZNF domain	639
PREX2	phosphatidylinositol-3,4,5-trisphosphate-dependent Rac exchange factor 2	80243
PRKAR1A	protein kinase, cAMP-dependent, regulatory, type I, alpha (tissue specific extinguisher 1)	5573
PRKCA	protein kinase C, alpha	5578
PRKCB	protein kinase C, beta	5579
PRKCG	protein kinase C, gamma	5582
PRKDC	similar to protein kinase, DNA-activated, catalytic polypeptide; protein kinase, DNA-activated, catalytic polypeptide	5591
PRLR	prolactin receptor	5618
PRMT5	protein arginine methyltransferase 5	10419
PROZ	protein Z, vitamin K-dependent plasma glycoprotein	8858
PRRX1	paired related homeobox 1	5396
PRSS1	protease, serine, 1 (trypsin 1); trypsinogen C	5644
PRX	periaxin	57716
PTCH1	patched homolog 1 (Drosophila)	5727
PTCH2	patched homolog 2 (Drosophila)	8643
PTEN	phosphatase and tensin homolog; phosphatase and tensin homolog pseudogene 1	5728
PTP4A1	protein tyrosine phosphatase type IVA, member 1	7803
PTPN11	protein tyrosine phosphatase, non-receptor type 11; similar to protein tyrosine phosphatase, non-receptor type 11	5781

PTPN22	protein tyrosine phosphatase, non-receptor type 22 (lymphoid)	26191
PTPRD	protein tyrosine phosphatase, receptor type, D	5789
PTPRN2	protein tyrosine phosphatase, receptor type, N polypeptide 2	5799
RABGEF1	RAB guanine nucleotide exchange factor (GEF) 1	27342
RAC1	ras-related C3 botulinum toxin substrate 1 (rho family, small GTP binding protein Rac1)	5879
RAD21	RAD21 homolog (S. pombe)	5885
RAD50	RAD50 homolog (S. cerevisiae)	10111
RAD51	RAD51 homolog (RecA homolog, E. coli) (S. cerevisiae)	5888
RAD51B	RAD51 paralog B	5890
RAD51C	RAD51 homolog C (S. cerevisiae)	5889
RAD51D	RAD51 paralog D	5892
RAD52	RAD52 homolog (S. cerevisiae)	5893
RAF1	v-raf-1 murine leukemia viral oncogene homolog 1	5894
RALA	RAS like proto-oncogene A	5898
RALB	RAS like proto-oncogene B	5899
RB1	retinoblastoma 1	5925
RBFOX1	RNA binding protein, fox-1 homolog (C. elegans) 1	54715
RBM10	RNA binding motif protein 10	8241
REG4	regenerating islet-derived family, member 4	83998
REL	v-rel reticuloendotheliosis viral oncogene homolog (avian)	5966
RET	ret proto-oncogene	5979
RHEB	Ras homolog enriched in brain	6009
RHOA	ras homolog family member A	387
RICTOR	RPTOR independent companion of MTOR, complex 2	253260
RIOK3	RIO kinase 3 (yeast)	8780
RIT1	Ras like without CAAX 1	6016
RNF19A	ring finger protein 19A	25897
RNF32	ring finger protein 32	140545
RNF43	ring finger protein 43	54894

ROBO1	roundabout, axon guidance receptor, homolog 1 (Drosophila); similar to roundabout 1 isoform b	6091
ROBO2	roundabout, axon guidance receptor, homolog 2 (Drosophila)	6092
ROS1	c-ros oncogene 1 , receptor tyrosine kinase	6098
RPS6KA5	ribosomal protein S6 kinase, 90kDa, polypeptide 5	9252
RPS6KB1	ribosomal protein S6 kinase, 70kDa, polypeptide 1	6198
RPS6KC1	ribosomal protein S6 kinase, 52kDa, polypeptide 1	26750
RPTN	repetin	126638
RPTOR	regulatory associated protein of MTOR, complex 1	57521
RSPO1	R-spondin homolog (Xenopus laevis)	284654
RSPO2	R-spondin 2 homolog (Xenopus laevis)	340419
RSPO3	R-spondin 3 homolog (Xenopus laevis)	84870
RSPO4	R-spondin family, member 4	343637
RUNX1	runt-related transcription factor 1	861
SAV1	salvador homolog 1 (Drosophila)	60485
SDK1	sidekick homolog 1, cell adhesion molecule (chicken); hypothetical LOC730351	221935
SELL	selectin L	6402
SETD2	SET domain containing 2	29072
SF3B1	splicing factor 3b, subunit 1, 155kDa	23451
SFRP1	secreted frizzled-related protein 1	6422
SGK1	serum/glucocorticoid regulated kinase 1	6446
SH2B3	SH2B adaptor protein 3	10019
SIM1	single-minded homolog 1 (Drosophila)	6492
SKP2	S-phase kinase associated protein 2	6502
SLIT2	slit homolog 2 (Drosophila)	9353
SMAD2	SMAD family member 2	4087
SMAD3	SMAD family member 3	4088
SMAD4	SMAD family member 4	4089
SMARCA1	SWI/SNF related, matrix associated, actin dependent regulator of chromatin, subfamily a, member 1	6594

SMARCA2	SWI/SNF related, matrix associated, actin dependent regulator of chromatin, subfamily a, member 2	6595
SMARCA4	SWI/SNF related, matrix associated, actin dependent regulator of chromatin, subfamily a, member 4	6597
SMARCA5	SWI/SNF related, matrix associated, actin dependent regulator of chromatin, subfamily a, member 5	8467
SMARCAD1	SWI/SNF-related, matrix-associated actin-dependent regulator of chromatin, subfamily a, containing DEAD/H box 1	56916
SMARCAL1	SWI/SNF related, matrix associated, actin dependent regulator of chromatin, subfamily a-like 1	50485
SMARCB1	SWI/SNF related, matrix associated, actin dependent regulator of chromatin, subfamily b, member 1	6598
SMARCC1	SWI/SNF related, matrix associated, actin dependent regulator of chromatin, subfamily c, member 1	6599
SMARCC2	SWI/SNF related, matrix associated, actin dependent regulator of chromatin, subfamily c, member 2	6601
SMARCD1	SWI/SNF related, matrix associated, actin dependent regulator of chromatin, subfamily d, member 1	6602
SMARCD2	SWI/SNF related, matrix associated, actin dependent regulator of chromatin, subfamily d, member 2	6603
SMARCD3	SWI/SNF related, matrix associated, actin dependent regulator of chromatin, subfamily d, member 3	6604
SMARCE1	SWI/SNF related, matrix associated, actin dependent regulator of chromatin, subfamily e, member 1	6605
SMO	smoothened homolog (Drosophila)	6608
SNTG2	syntrophin, gamma 2	54221
SNX31	sorting nexin 31	169166
SOX17	SRY (sex determining region Y)-box 17	64321

SOX2	SRY (sex determining region Y)-box 2	6657
SOX9	SRY-box 9	6662
SPEN	spen homolog, transcriptional regulator (Drosophila)	23013
SPOP	speckle-type POZ protein	8405
SPRY1	sprouty homolog 1, antagonist of FGF signaling (Drosophila)	10252
SPRY2	sprouty homolog 2 (Drosophila)	10253
SPRY3	sprouty homolog 3 (Drosophila)	10251
SPRY4	sprouty homolog 4 (Drosophila)	81848
SS18	synovial sarcoma translocation, chromosome 18	6760
STAG2	stromal antigen 2	10735
STAT3	signal transducer and activator of transcription 3 (acute-phase response factor)	6774
STK11	serine/threonine kinase 11	6794
STK19	serine/threonine kinase 19	8859
STK3	serine/threonine kinase 3 (STE20 homolog, yeast)	6788
STRADA	STE20-related kinase adaptor alpha	92335
STXBP5L	syntaxin binding protein 5-like	9515
SUFU	suppressor of fused homolog (Drosophila)	51684
TAB3	mitogen-activated protein kinase kinase kinase 7 interacting protein 3	257397
TACC1	transforming, acidic coiled-coil containing protein 1	6867
TACC3	transforming, acidic coiled-coil containing protein 3	10460
TBC1D7	TBC1 domain family, member 7	51256
TBX3	T-box 3	6926
TCF7	transcription factor 7 (T-cell specific, HMG-box)	6932
TCF7L1	transcription factor 7-like 1 (T-cell specific, HMG-box)	83439
TCF7L2	transcription factor 7-like 2 (T-cell specific, HMG-box)	6934
TEAD4	TEA domain transcription factor 4	7004
TERT	telomerase reverse transcriptase	7015
TET2	tet oncogene family member 2	54790

TEX15	testis expressed 15	56154
TGFBR2	transforming growth factor, beta receptor II (70/80kDa)	7048
TLR3	toll-like receptor 3	7098
TMEM173	transmembrane protein 173	340061
TMPRSS2	transmembrane protease, serine 2	7113
TNFSF10	tumor necrosis factor (ligand) superfamily, member 10	8743
TNKS	tankyrase, TRF1-interacting ankyrin-related ADP-ribose polymerase	8658
TNKS2	tankyrase, TRF1-interacting ankyrin-related ADP-ribose polymerase 2	80351
TOP1	topoisomerase (DNA) I	7150
TP53	tumor protein p53	7157
TP63	tumor protein p63	8626
TP73	tumor protein p73	7161
TPK1	thiamin pyrophosphokinase 1	27010
TPR	translocated promoter region (to activated MET oncogene)	7175
TRAF2	TNF receptor-associated factor 2	7186
TRAF3	TNF receptor-associated factor 3	7187
TRAF7	TNF receptor-associated factor 7	84231
TRRAP	transformation/transcription domain-associated protein	8295
TSC1	tuberous sclerosis 1	7248
TSC2	tuberous sclerosis 2	7249
TSHR	thyroid stimulating hormone receptor	7253
TSTD1	thiosulfate sulfurtransferase KAT, putative	100131187
TTK	TTK protein kinase	7272
TUBD1	tubulin, delta 1	51174
U2AF1	U2 small nuclear RNA auxiliary factor 1	7307
VEGFA	vascular endothelial growth factor A	7422
VHL	von Hippel-Lindau tumor suppressor	7428
VPREB1	pre-B lymphocyte 1	7441
WDFY4	WDFY family member 4	57705
WDR5	WD repeat domain 5	11091
WHSC1	Wolf-Hirschhorn syndrome candidate 1	7468
WHSC1L1	Wolf-Hirschhorn syndrome candidate 1-like 1	54904
WRN	Werner syndrome RecQ like helicase	7486

WSB1	WD repeat and SOCS box-containing 1	26118
WT1	Wilms tumor 1	7490
WWOX	WW domain containing oxidoreductase	51741
YAF2	YY1 associated factor 2	10138
YAP1	Yes-associated protein 1, 65kDa	10413
ZC3H13	zinc finger CCCH-type containing 13	23091
ZEB2	zinc finger E-box binding homeobox 2	9839
ZNF132	zinc finger protein 132	7691
ZNF217	zinc finger protein 217	7764
ZNF324	zinc finger protein 324	25799
ZNF639	zinc finger protein 639	51193
ZNF703	zinc finger protein 703	80139
ZNF704	zinc finger protein 704	619279
ZNF704	zinc finger protein 704	619279

Supplementary Table 3: Selected introns

Gene Symbol	Gene Description	Entrez Gene ID
ALK	anaplastic lymphoma receptor tyrosine kinase	238
BRAF	B-Raf proto-oncogene, serine/threonine kinase	673
BRD4	bromodomain containing 4	23476
CTNNB1	catenin (cadherin-associated protein), beta 1, 88kDa	1499
EGFR	epidermal growth factor receptor	1956
EGFR	epidermal growth factor receptor	1956
ELK4	ELK4, ETS-domain protein (SRF accessory protein 1)	2005
EML4	echinoderm microtubule associated protein like 4	27436
ERG	v-ets avian erythroblastosis virus E26 oncogene homolog	2078
ETV1	ets variant 1	2115
ETV4	ets variant 4	2118
ETV6	ets variant 6	2120
EWSR1	EWS RNA-binding protein 1	2130
FGFR1	fibroblast growth factor receptor 1	2260
FGFR2	fibroblast growth factor receptor 2	2263
FGFR3	fibroblast growth factor receptor 3	2261
FGFR4	fibroblast growth factor receptor 4	2264
MET	MET proto-oncogene, receptor tyrosine kinase	4233
NOTCH1	notch 1	4851
NTRK1	neurotrophic tyrosine kinase, receptor, type 1	4914
PAX3	paired box 3	5077
RAF1	Raf-1 proto-oncogene, serine/threonine kinase	5894
RET	ret proto-oncogene	5979
ROS1	ROS proto-oncogene 1 , receptor tyrosine kinase	6098
RSPO2	R-spondin 2	340419
RSPO3	R-spondin 3	84870
SUZ12	SUZ12 polycomb repressive complex 2 subunit	23512
TFE3	transcription factor binding to IGHM enhancer 3	7030

TMPRSS2	transmembrane protease, serine 2	7113
VTI1A	vesicle transport through interaction with t-SNAREs 1A	143187

Supplementary Table 4:

**Specimen Type**	**Case 1**	**Case 2**	**Case 3**	**Case 4**	**Case 5**	**Case 6**	**Case 7**	**Case 8**	**Case 9**
Normal	X	X	X	X	X	X	X	X	X
Tumor	X	-	X	X	-	X	X	X	X
Paired Non-tumor	X	-	X	X	-	X	X	X	X
Blood	X	X	X	X	X	-		X	X
Pleural Effusion	-	X	X	-	-	-	X	-	X
Pericardial fluid	-	-	-	X	-	-	-	-	-

Supplementary Table 5. Summary of numbers of samples with tumor after evaluation of hematoxylin and eosin slides from formalin-fixed paraffin embedded blocks. “Primary lung” samples were collected from the first site diagnosed with lung cancer and “secondary lung” samples were collected from any later sites separated from the primary site that were identified with cancer.

**Tumor Site**	**Number of Samples with Tumor Confirmed Microscopically**									
	Case 1	Case 2	Case 3	Case 4	Case 5	Case 6	Case 7	Case 8	Case 9	**Total**
**Primary Lung**	1	0	1	1	0	1	1	1	1	**7**
**Secondary Lung**	1	0	2	1	0	0	0	3	1	**8**
**Pericardium**	0	0	0	0	0	0	2	0	0	**2**
**Liver**	1	0	0	1	0	0	0	0	4	**6**
**Kidney**	2	0	0	0	0	0	0	0	0	**2**
**Lymph Nodes**	2	0	0	1	0	1	1	1	0	**6**
**Adrenal Gland**	1	0	0	1	0	0	0	1	0	**3**
**Bone**	0	0	0	0	0	0	0	3	0	**3**
**Total**	**8**	**0**	**3**	**5**	**0**	**2**	**4**	**9**	**6**	**37**

Supplementary Table 6:

Study ID	Anatomic Location	Protocol	Estimated Tumor Purity	Mean Target Coverage	PCT Selected Bases	# coding SNV	# unique SNV	Oncogene	Estimated Library Size (millions)	PCT Coding	PCT Duplication
1	Left Lung	DNA	0.16	481.4	87.3%	14	1	KRAS G12V 14%			
1	Liver	DNA	0.29	481.3	87.0%	20	0	KRAS G12V 51%			
1	Mediastinal Lymph Node	DNA	0.2	448.7	87.6%	21	1	KRAS G12V 19%			
1	Right Lung	DNA	0.24	609.3	89.3%	21	1	KRAS G12V 41%			
3	Lung Right Lower Lobe	DNA	0.55	547.4	88.7%	12	1	KRAS G12V 55%			
3	Lung Right Upper Lobe	DNA	0.73	731.6	84.6%	14	2	KRAS G12V 49%			
4	Liver	DNA	0.55	554.8	88.0%	15	2	KEAP1 E441* 45%, TP53 N239D 41%			
4	Pericardinal Lymph Node	DNA	0.66	528.8	87.0%	13	0	TP53 N239D 79%			
4	Right Lung	DNA	0.8	472.4	86.1%	16	2	KEAP1 E441* 87%, TP53 N239D 80%			
6	Lung Left Lower Lobe	DNA	0.17	537.9	88.6%	7	1	BRCA2 R2034C 50%, KRAS G12D 3%			
6	Lymph Node	DNA	0.16	632.2	87.5%	20	14	BRCA2 R2034C 44%, KRAS G12D 10%			
7	Lung Left Hilar Region	DNA	0.3	817.2	89.0%	10	1	SMARCA4 R1135Q 13%			
7	Mediastinal Lymph Node	DNA	0.16	638.0	88.7%	9	0	SMARCA4 R1135Q 6%			
1	Left Lung	RNA							210.64	13.9%	9%
1	Liver	RNA							88.30	16.7%	15%
1	Mediastinal Lymph Node	RNA							29.38	13.6%	31%
1	Right Lung	RNA							82.13	15.4%	16%
3	Lung Right Lower Lobe	RNA							216.29	14.5%	9%
3	Lung Right Upper Lobe	RNA							125.94	13.8%	12%
4	Liver	RNA							147.56	18.1%	12%
4	Pericardinal Lymph Node	RNA							89.58	11.6%	15%
4	Right Lung	RNA							15.07	11.7%	49%
6	Lung Left Lower Lobe	RNA							286.98	16.1%	8%
6	Lymph Node	RNA							186.74	17.0%	11%
7	Lung Left Hilar Region	RNA							249.41	19.3%	9%
7	Mediastinal Lymph Node	RNA							34.51	17.9%	28%

Supplementary table 7:

	Gene Symbol	Amino Acid Change	Lung Left	Lung Right	Mediastinal Lymph Node	Liver
Participant 1	PDCD1	F63V	0.037656904	0.067615658	0.072916667	0.156164384
	EPHA6	P1052Q	0.072929543	0.139393939	0.119422572	0.219736842
	SMARCA5	D151Y	0.058295964	0.217228464	0.094562648	0.341708543
	FAT1	G1707S	0.054363376	0.196078431	0.122866894	0.305555556
	JAK2	K558N	0.472380952	0.546052632	0.45561139	0.480662983
	ATM	R2832L	0.058823529	0.087096774	0.0625	0.032840722
	KRAS	G12V	0.13553719	0.405870445	0.192125984	0.513872135
	PARP4	V1111M	0.054325956	0.061749571	0.065263158	0.053452116
	PIF1	V172M	0.494163424	0.475570033	0.502347418	0.474777448
	PRKCB	P643T	0.052991453	0.126631854	0.092391304	0.263461538
	TP53	K132N	0.049180328	0.237442922	0.12	0.304545455
	CDK12	E281D	0.070093458	0.099667774	0.07254902	0.171875
	EIF1AX	G6V, Splice site	0.054320988	0.084775087	0.074235808	0.059090909
	ARID1B	A852V	0.03962704			
	PTPRD	Splice site		0.064516129	0.076246334	0.274052478
	ATOH7	H115D		0.221052632	0.079646018	0.290697674
	FAT3	D2950Y		0.125944584	0.088679245	0.20625
	BCOR	C94G		0.164179104	0.079295154	0.191428571
	ARAF	E354K		0.144144144	0.115789474	0.215189873
	ATRX	K1429N		0.131406045	0.059504132	0.203767123
	STXBP5L	R789*			0.1015625	

	Gene Symbol	Amino Acid Change	Lung Right Lower Lobe	Lung Right Upper Lobe
Participant 3	STK11	G268 frameshift	0.82175926	0.8742515
	TET2	P1723S	0.57495591	0.41356383
	KRAS	G12V	0.55459459	0.48764259
	BRCA1	S1165*	0.53673724	0.59250399
	PCDH15	R275C	0.51031637	0.48028311
	FOXC1	A493V	0.50655022	0.61971831
	DZIP1	G112V	0.47407407	0.43373494
	GRIN2A	V187F	0.44736842	0.39798489
	ERBB3	T478S	0.43067847	0.43783784
	PIK3CA	R662M	0.37818182	0.39713542
	DCC	G276C	0.36726547	0.2729927
	ZNF324	G525 frameshift	0.22527473	0.36190476
	NTRK3	R694T	0.02571429	0.1847619
	MECOM	G610W	0.15069968	
	MECOM	G610E frameshift	0.14412636	
	FBXW7	Splice site indel	0.06849315	
	NOTCH4	Splice site		0.14338235
	PIK3R4	Splice site indel		0.05128205
	STAG2	Splice site indel		0.06896552

	Gene Symbol	Amino Acid Change	Lung Right	Pericardinal Lymph Node	Liver
Participant 4	RB1	Y805 frameshift	0.856115108	0.717285945	0.355430183
	SMARCA4	R973L	0.805555556	0.720496894	0.48
	MSH3	P231S	0.803921569	0.778350515	0.302120141
	TP53	N239D	0.801104972	0.786458333	0.412765957
	NTRK3	I321N	0.777777778	0.701754386	0.438016529
	FGF4	Y146*	0.606382979	0.509259259	0.542553191
	RBM10	P388S	0.47706422	0.552995392	0.497560976
	AR	E255Q	0.473684211	0.447674419	0.2109375
	PRSS1	G143V	0.443946188	0.393305439	0.287037037
	SF3B1	Y412C	0.442913386	0.40173913	0.227474151
	KDM5C	D1430E	0.427230047	0.408376963	0.224043716
	AVPR1A	A273S	0.364963504	0.411027569	0.250554324
	PARD6G	L360V	0.299435028	0.338461538	0.377906977
	AFF2	L199V	0.466284075	0.329896907	
	KEAP1	E441*	0.867647059		0.446153846
	PPP1R3A	L589F			0.036637931
	PCSK5	A1451D			0.16909621
	ATRX	T785I	0.139851485		

	Gene Symbol	Amino Acid Change	Lung Left Lower Lobe	Lymph Node
**Participant 6**	BRCA2	R2034C	0.498618785	0.439482961
	PDK1	M289I	0.458823529	0.488745981
	NTRK1	L316R	0.052427184	0.135061392
	KRAS	R123G	0.032	0.128550075
	KRAS	G12D	0.035117057	0.103040541
	PTPRD	A631S	0.028776978	0.063829787
	EPHA3	D144V		0.065194532
	MECOM	NS		0.062972292
	MECOM	NS		0.062972292
	FLT4	G541C		0.111111111
	PHF3	E763*		0.080931264
	HGF	T48N		0.071535022
	TRRAP	S1503A		0.096
	DLGAP2	L891H		0.057803468
	PTPRD	V687L		0.091286307
	KLF4	A67S		0.089285714
	TCF7L2	D181A		0.072727273
	FAT3	V586D		0.098258706
	FAT3	Q3198H		0.057352941
	FLT1	*734L		0.055984556
	RBM10	E605*		0.170616114
	FBXW7	Splice site indel		

	Gene Symbol	Amino Acid Change	Lung Left Hilar Region	Mediastinal Lymph Node
**Participant 7**	KMT2D	G1234R	0.48854962	0.51567944
	CYP7B1	A224S	0.47129506	0.4641791
	SYTL2	A95T	0.47058824	0.50980392
	ACVR1B	A35S	0.45792079	0.48517941
	HRNR	S840T	0.44217687	0.44354839
	HRNR	S2731L	0.1559633	0.0952381
	SMARCA4	R1135Q	0.13274336	0.06321839
	LONRF1	L584F	0.12953368	0.06952965
	CDH5	T127S	0.09253731	0.05990783
	EML4-ALK	translocation	0.0282023	0.0272473
	BRAF-AGK	translocation	0.02668684	0.02799543
	CDH5	E224Q	0.08284024	
	EZH2	Splice site indel	0.07407407	
