# Supplementary material for: A community‐based lung cancer rapid tissue donation protocol provides high‐quality drug‐resistant specimens for proteogenomic analyses
Source: Cancer Med. 2019 Nov 20;9(1):225–37. doi: 10.1002/cam4.2670 (PMC6943158; doi:10.1002/cam4.2670)
Supplement: Supplementary file 3 [file CAM4-9-225-s003.pdf]

Supplementary Document 1: Antibody Information

- PD-L1 rabbit monoclonal antibody clone E1L3N® (Cell Signaling Technologies, Danvers, MA)[9]
- PD-L1 rabbit monoclonal antibody, clone 28-8 (Agilent Dako, Carpinteria, CA, USA)
- Ki67 (anti-Ki67 rabbit monoclonal antibody, clone 30-9,
- CD8 (anti-CD8 mouse monoclonal antibody, clone C8/144B, Agilent Dako, Carpinteria, CA, USA), Roche Ventana, Tucson, AZ, USA),
- CD31 (anti-CD31 rabbit monoclonal antibody, ab28364, Abcam, Cambridge, MA, USA)
- pSTAT3 (anti-phospho-STAT3 rabbit monoclonal antibody, clone 58E12, Cell Signaling Technology, Danvers, MA, USA

Supplementary Document 2: Additional Sequencing Information

Agilent ClearSeq Comprehensive Cancer Panel:

Methods: Next generation sequencing (NGS) was performed on 11 DNA samples extracted from frozen tumor tissue using the Agilent ClearSeq Comprehensive Cancer panel protocol.[1] Sequence reads were aligned with BWA and refined with GATK and PICARD (http://picard.sourceforge.net/). Variants were detected using GATK and enriched for somatic mutations by filtering against 1000 Genomes and Exome Sequencing Project (ESP). Variants were annotated by ANNOVAR. Fusions were detected using Manta software.

Illumina Trusight Tumor 170 (TST170):

Methods: Library preparation was performed using the hybrid capture-based TruSight Tumor 170 Library Preparation Kit (Illumina, San Diego, CA) following the manufacturer's protocol: TruSight Tumor Reference Guide available at: https://support.illumina.com. Libraries were sequenced on a NextSeq 500 or 550 (Illumina), with 16 libraries (8 DNA and 8 RNA) sequenced per run to achieve maximum sample coverage. The sequence data was processed through the BaseSpace TruSight Tumor 170 App (Illumina) with creation of BCL files and generation of fastQ files. Results from the App were subsequently transferred and reported with CGW software from Washington University. The filter for fusions was based on the "high confidence" filter in the TST170 BaseSpace App which uses a Mantra-based algorithm.

References:

1. Technologies A. ClearSeq Comprehensive Cancer Next Generation Comprehensive Cancer Profiling. *NGS Disease Research Panels.* 2016.

Supplementary Document 3: The average of read counts from a cell cycle signature of 19 genes

RNAseq cell cycle signature of 19 genes:

AURKB, *BIRC5, BUB1B, CCNA2, CCNB1, CCNB2, CCNE1, CDC6, CDC7, CDC20, CENPE, CENPF, KIF2C, KIF20A, MAD2L1, MKI67, NDC80, PTTG1, TOP2A*

RNAseq liver specific signature of 21 genes:

HPX, FGA, AMBP, FGG, FGB, ALB, CYP2C8, ORM1, CRP, FGL1, CYP2E1, GC, AGXT, APOH, MAT1A, TF, AHSG, HRG, APOA2, HP, RBP4, and ADH4
